# Landscape of the Dark Transcriptome Revealed Through Re-mining Massive RNA-Seq Data

**DOI:** 10.3389/fgene.2021.722981

**Published:** 2021-08-16

**Authors:** Jing Li, Urminder Singh, Zebulun Arendsee, Eve Syrkin Wurtele

**Affiliations:** ^1^Genetics and Genomics Graduate Program, Iowa State University, Ames, IA, United States; ^2^Department of Genetics, Development, and Cell Biology, Iowa State University, Ames, IA, United States; ^3^Center for Metabolic Biology, Iowa State University, Ames, IA, United States; ^4^Bioinformatics and Computational Biology Program, Iowa State University, Ames, IA, United States

**Keywords:** orphan gene, *de novo*, RNA-Seq, Ribo-seq, gene function, cluster analysis

## Abstract

The “dark transcriptome” can be considered the multitude of sequences that are transcribed but not annotated as genes. We evaluated expression of 6,692 annotated genes and 29,354 unannotated open reading frames (ORFs) in the *Saccharomyces cerevisiae* genome across diverse environmental, genetic and developmental conditions (3,457 RNA-Seq samples). Over 30% of the highly transcribed ORFs have translation evidence. Phylostratigraphic analysis infers most of these transcribed ORFs would encode species-specific proteins (“orphan-ORFs”); hundreds have mean expression comparable to annotated genes. These data reveal unannotated ORFs most likely to be protein-coding genes. We partitioned a co-expression matrix by Markov Chain Clustering; the resultant clusters contain 2,468 orphan-ORFs. We provide the aggregated RNA-Seq yeast data with extensive metadata as a project in MetaOmGraph (MOG), a tool designed for interactive analysis and visualization. This approach enables reuse of public RNA-Seq data for exploratory discovery, providing a rich context for experimentalists to make novel, experimentally testable hypotheses about candidate genes.

## Introduction

**FIGURE 1:**
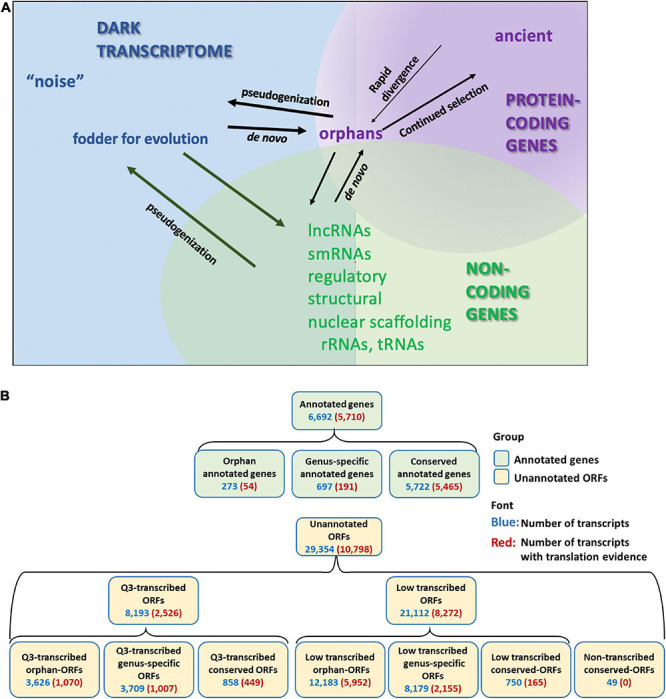
Annotated genes and dark transcriptome. **(A)** Definition of Dark transcriptome. Pervasive transcription of unannotated sequences has been found in many species. Some of these might be protein coding genes that have escaped annotation. Most of these unannotated coding genes are orphan (species-specific) genes, which have no homolog to other species, and are hard to predict using current gene prediction tools. These orphan genes could emerge by rapid divergence from ancient genes or could evolve *de novo*. Other transcribed but unannotated sequences might be non-coding genes. Although many studies have explored the function and classification of the non-coding transcripts, many transcribed sequences are still unclassified. **(B)** Classification and numbers of transcripts with transcription or translation evidence for annotated genes and open reading frames (ORFs). Orphan-ORFs, protein is unique to *Saccharomyces cerevisiae* [phylostrata (PS) = 15]; genus-specific-ORFs, protein is unique to *Saccharomyces* spp. (PS = 10–14); conserved-ORFs, protein has homologs in older species (PS = 1–9). Q3-transcribed, ORFs with mean expression values across the 3,457 samples in the upper (Q3) quantile of the unannotated transcripts. Low-transcribed ORFs, ORFs with mean expression values across the 3,457 samples in the lower 75% quantile of the unannotated transcripts. Non-transcribed conserved-ORFs, no transcription evidence detected (Supplementary Figure 8). (For full PS designations and transcription expression, see Supplementary Material, *S. cerevisiae*_RNA-seq_3457_27.mog; for translation per transcript, see Supplementary Material, *Ribo-Seq_rawcounts.csv.*).

Pervasive transcription of unannotated genome sequence in eukaryotic species is evidenced in multiple RNA-Seq studies (Struhl, 2007; Hangauer et al., 2013; Lu et al., 2017; Pertea et al., 2018; Wu and Knudson, 2018). Indeed, transcription and translation has been described for non-genic regions of genomes in diverse species (Wilson and Masel, 2011; Carvunis et al., 2012; Chew et al., 2013; Ruiz-Orera et al., 2014, 2015; Smith et al., 2014; Hsu et al., 2016; Prabh and Rödelsperger, 2016; Olexiouk et al., 2017; Choudhary et al., 2019). Many studies have dismissed this expression as transcriptional “noise” (ENCODE Project Consortium, 2012; Lloréns-Rico et al., 2016; Barroso et al., 2018; Pertea et al., 2018). However, functional genes have been identified from the so-called “noise” (Andrews and Rothnagel, 2014; Ji et al., 2015). This mass of unannotated transcripts, often ignored and little understood, we refer to as the “dark transcriptome” (Figure 1A).

Each organism contains species-specific genes (denoted here as “orphan genes”). The challenge of distinguishing orphan genes in genomes and predicting their functions is immense, resulting in an under-appreciation of their importance. The emergence of novel protein coding genes specific to a single species (orphans) is a vital mechanism that allows organisms to survive a changing environment (Domazet-Lošo et al., 2007; Tautz and Domazet-Lošo, 2011; Carvunis et al., 2012; Arendsee et al., 2014; Schlötterer, 2015; McLysaght and Hurst, 2016). Over generations, those orphan genes that continue to provide a survival advantage will be maintained. Orphan genes can be identified from within a list of genes by phylostratigraphy, the classification of each gene according to its inferred age of emergence (Domazet-Lošo et al., 2007; Tautz and Domazet-Lošo, 2011). Two general mechanisms enable orphan gene emergence:(1) *de novo* evolution and (2) divergence of proteins of existing genes beyond recognition in a short time frame.

Orphan genes can evolve *de novo* from non-coding sequence in regions of the genome lacking genes entirely or as new reading frames within existing genes (Tautz and Domazet-Lošo, 2011; Ruiz-Orera et al., 2015; Arendsee et al., 2019a; Van Oss and Carvunis, 2019). Indeed, transcriptional and translational “noise” has been suggested as a mechanism that facilitates novel gene emergence (Chen et al., 2013; Hoen and Bureau, 2015; Landry et al., 2015; Gubala et al., 2017; Ruiz-Orera et al., 2018; Xie et al., 2019). This hypothesis is borne out by *in vitro* and *in vivo* synthetic biology research demonstrating that novel peptides are often able to bind small molecules (e.g., ATP, and metals) (Bao et al., 2017) and induce beneficial phenotypes when expressed (Bao et al., 2017; Neme et al., 2017). If information on the expression of the dark transcriptome was more easily accessible, the potential roles of expressed transcripts could be better considered.

Orphan genes can also evolve from existing proteins by divergence of protein coding sequences (CDSs) beyond recognition (Khalturin et al., 2009; Tautz and Domazet-Lošo, 2011; Chen et al., 2013; Menschaert et al., 2013; Vanderperre et al., 2013; Landry et al., 2015; Gubala et al., 2017; Xie et al., 2019; Vakirlis et al., 2020). We estimate from the phylostratigraphic data on yeast genes that this process would require ultra-rapid sequence divergence relative to that of the average protein. Evolution of orphan genes from existing protein-coding genes has been estimated to account for about 18% (human), 25% (Drosophila), and 45% (yeast) of annotated taxonomically restricted genes (Vakirlis et al., 2020). However, this estimate can consider only the that can be compared across species, i.e., those that are located within syntenic intervals of related genomes; about ∼50% of genes for yeast (Arendsee et al., 2019b).

A systematic analysis of current computational methods for genome annotation indicates many orphan genes may be missed in annotation projects (Li et al., 2021). This is because genes are often identified from sequenced genomes by combining evidence based on homology with other species (Meyer and Durbin, 2004; Proux-Wéra et al., 2012) with *ab initio* machine-learning predictions by detecting canonical sequence motifs (e.g., splice junctions) (Cantarel et al., 2008; Hoff et al., 2016). However, homology and *ab initio* approaches can be problematic in predicting orphan genes. First, orphan genes cannot be identified by homology to genes of other species, since they have none. Secondly, to the extent that an orphan has not yet evolved canonical motifs, *ab initio* prediction may be ineffective (Li et al., 2021). For example, compared to the gold-standard annotations in the curated TAIR community database (Berardini et al., 2015), the popular *ab initio* pipeline *MAKER* (Cantarel et al., 2008) predicted as few as 11% of the annotated *Arabidopsis* orphan genes, depending on the RNA-Seq evidence supplied (Li et al., 2021). Enhancing *ab initio* pipelines by other sequence-based information [e.g., motif/domain information, cellular location predictions, predicted isoelectric point (pI), and genomics context] can improve gene predictions (Grandaubert et al., 2015; González et al., 2016; Werner et al., 2018).

However, because it is not a given that newly evolved genes have canonical features, direct alignment of transcriptomic and/or proteomic data to the genome is critical for annotating orphan genes, as well as non-coding transcripts (lncRNAs, etc.) (Carvunis et al., 2012; Ruiz-Orera et al., 2014, 2018; González et al., 2016; Lu et al., 2017; Wu and Knudson, 2018; Li et al., 2021; Blevins et al., 2021).

Here, we reuse and re-mine aggregated RNA-Seq data to discover new potential gene candidates. The study comprehensively evaluates transcription and ribosomal binding of all open reading frames (ORFs) in the yeast genome over a wide variety of conditions, in the context of annotated genes. The research extends the results of previous studies, in that it globally represents ORFs in the *Saccharomyces cerevisiae* genome across thousands of samples. Furthermore, we provide these data and extensive metadata via a biologist-friendly platform, MetaOmGraph (MOG; Singh et al., 2020),^1^ which provides interactive, exploratory analysis (Tukey, 1977) and visualization of expression levels, expression conditions, and co-expressed genes for the ORF-containing transcripts. This approach enables experimentalists to prioritize ORFs for functional characterization, and to logically define experimental parameters for these characterizations (Singh et al., 2020).

## Materials and Methods

### Extracting ORFs and Delineating Orphan-ORFs in *Saccharomyces cerevisiae*

In order to evaluate potential ORFs in the yeast genome comprehensively, all ORFs over 150 nt were extracted from the yeast genome (version: R64-1-1) by *emboss getorf* (v6.6.0) using “-minsize 150” and “-find 3,” and translated by *emboss transeq* (Rice et al., 2000). Then, we removed the ORFs identical to annotated genes in Saccharomyces Genome Database (SGD) based on coordinates by *bedtools2* (Quinlan and Hall, 2010). We further removed ORFs within annotated genes in same translation frame. These filtrations yielded 24,912 ORFs. To these ORFs we added two sets of ORFs < 150 nt identified in other studies: the 1,139 small translated sequences (smORFs) identified by ribosome profiling (Carvunis et al., 2012) and the 3,303 of ORFs identified by TIF-Seq (txCDS; Lu et al., 2017) that were less than 150 nt (thus, not included in the EMBOSS extraction). These 29,354 ORFs, together with the 6,692 protein-coding genes annotated in SGD, were subjected to phylostratigraphic analysis.

We inferred the phylostratum for 29,354 ORFs and 6,692 annotated protein-coding genes via the R package, *phylostratr* (v0.2.0) (Arendsee et al., 2019b). This analysis compared the proteins predicted from the cORFs to UniProt-annotated proteins of 123 target species distributed across phylostrata: 117 species were identified by the *phylostratr* algorithm; these were supplemented with six manually selected species in the *Saccharomyces* genus (*S. paradoxus, S. mikatae, S. kudriavzevii, S. arboricola, S. eubayanus*, and *S. uvarum*). To minimize false positives when identifying orphan ORFs and CDS from *S. cerevisiae*, we took advantage of the customization capabilities of *phylostratr* to include the predicted translation products from all ORFs (>150 nt) from each of the six *Saccharomyces* genomes, in addition to all annotated proteins of these species [see Supplementary Figure 1 for workflow, Supplementary Section 12 for full species list, and phylostratr_heatmap.pdf for a gene by gene (and ORF by ORF) inference heatmap]. Each gene was assigned to the most evolutionarily-distant phylostratum that contains an inferred homolog based on the adjusted *p*-value (0.001 as cutoff) as calculated from *e*-value of BLASTP (blast-plus v2.11.0). A gene or ORF was inferred to be an orphan if its encoded protein was assigned the phylostratum level *S. cerevisiae*.

### Raw Read Processing and Network Optimization

Our RNA-Seq data analysis pipeline is shown in Supplementary Figure 2. We selected all samples with *S. cerevisiae* taxon ID 4932, Illumina platform, and paired layout from The National Center for Biotechnology Information-Sequence Read Archive (NCBI-SRA) and then filtered out samples with miRNA-Seq, ncRNA-Seq, or RIP-Seq library strategies. In total, we collected raw reads data (FASTQ format) and metadata from 3,457 RNA-Seq samples (177 studies). A kallisto index was created from a FASTA file combining the cDNAs of annotated genes and unannotated ORFs (Weijers et al., 2012) with default setting (kallisto index -i yeast.allcdna allcdna.fasta), and expression levels of annotated genes and ORFs over the 3,457 RNA-Seq samples were quantified by *kallisto* (v0.43.1) with the bootstrap option “-b 100” and other default settings [Kallisto with and without bias option give similar accuracy (Bray et al., 2016), we used the default setting without bias correction] (see Supplementary Material S.cerevisiae_RNA-seq.mog for RNA-Seq metadata and normalized cpm data; all data including raw counts is accessible at DataHub)^2^.

Strand-specific libraries provide accurate determination of sense vs. antisense transcription (Zhao et al., 2015), however, most yeast RNA-Seq data was non-stranded. We quantified all of the yeast RNA-Seq samples available on NCBI-SRA using the “unstranded” option. Then, we quantified the 5% (177 samples) of the available RNA-Seq samples are strand-specific using the strandness option. We compared the expression of each annotated and unannotated transcript as quantified by specific strandness (“–fr-stranded” or “–rf-stranded”) with the expression of each annotated and unannotated transcript using the default option (no strand-specific) for the 177 samples. Then, we examined the pairwise Pearson correlation according to the expression with and without strand-specific option for each sample, the correlations had a median of 0.85 (range 0.71–0.91; Supplementary Figure 3). These high correlations between gene expression in the unstranded mode and stranded mode are consistent with the estimation for the unstranded RNA-Seq samples having little effect on our downstream Pearson correlation-based clustering analysis.

We normalized raw counts by *edgeR* (v3.22.3) (Robinson et al., 2010) based on the evaluation (Dillies et al., 2013), and evaluated the performance of normalization by comparing to the raw counts (Supplementary Section 3). After normalization, We further defined the robustly expressed ORFs, as defined by mean expression values in the upper quantile (Q3-transcribed ORFs). This subset of ORFs were used in some of the analyses, in particular the network analysis in which consideration of sparsely expressed and low-expressed transcripts are problematic. In subsequent methods and results, if “Q3-transcribed” is not designated, all transcribed ORFs were used in an analysis.

We generated two datasets: (1) all annotated genes (SGD dataset); and (2) all Q3-transcribed ORFs, smORFs, and annotated genes (SGD + ORF dataset). For each normalization approach and dataset, we calculated pairwise Pearson correlation matrices among all 3,457 RNA-Seq samples.

Three positive Pearson Correlation Coefficient (PCC) cutoffs, 0.6, 0.7 and 0.8, were used to create networks of different densities (Supplementary Section 4). We then applied Markov Cluster (MCL) algorithm to partition each network using our in-house Java Spark implementation (GitHub:^3^ designed to optimize efficiency. All data analysis in this work, except for MCL clustering and RNA-Seq expression visualization, were performed in R software (v3.5.0).

### Cluster Evaluation by GO Term Enrichment Analysis

Clusters resulting from each of the eight MCL analyses obtained from the different normalization methods and PCCs were evaluated by Gene Ontology (GO) enrichment analysis using *clusterProfiler* (v3.12.0) (Yu et al., 2012); only clusters with over five genes were considered. The GO term enrichment of each experimental result was compared to that of 100 random sets of clusters, which were obtained by permuting gene IDs. For these permutations, the same number and size of clusters as those from the experimental result were assigned to random sets using the method of Mentzen and Wurtele (2008). The best adjusted *p*-value (p_min_, smallest adjusted *p*-value) was recorded for the enriched GO terms in each cluster. Each random cluster set was assigned a score Si, which is the average p_min_ across all clusters in the set

(1) $$S_{i}=\frac{\sum_{j=1}^{n}p_{\min j}}{n}$$

where n indicates the number of clusters. The distribution of *S* values for GO classes, biological process (BP), cellular component (CC), and molecular function (MF), for random sets were compared to the respective values for the real experimental data. In each ontology, the experimental score was less than any of the random scores, indicating that experimental data have biological significance (permutation test, *p*-value = 0). Based on these GO enrichment results, we chose positive PCC = 0.6 as cutoff (Supplementary Section 4) for future analyses.

### Ribo-Seq Analysis

To investigate the translational activity of unannotated ORFs, we analyzed 302 samples (23 studies) of yeast Ribo-Seq data; this represented about half of the available Ribo-Seq in the SRA database. Raw reads (SRA-formatted) were downloaded, and the SRA toolkit was used to convert the raw reads to a FASTQ format. *BBDuk* (v38.75) was used to find and remove adapter sequences from the 3′ end of reads, and rRNA reads were identified and removed using *BBMap* (v38.75) with default option (Bushnell, 2014). The cleaned Ribo-Seq reads were aligned to the reference genome by *HISAT2* (Kim et al., 2015). The actively translating ORFs were detected and quantified by *Ribotricer* (v1.3.2), which considers the periodicity of ORF profiles and provides multiple options for customization (we used the recommended phase-score cutoff 0.318 for yeast) (Choudhary et al., 2019). The genes/ORFs with mean counts across 302 Ribo-Seq samples higher than 1.83 (This is the maximum mean counts for non-transcribed ORFs. According to Supplementary Figure 13, the Ribo-Seq expression for non-transcribed ORFs is too low so that we regard those expression lower than 1.83 as sequencing noise.) was consider to have translation evidence.

### Visualization and Gene Function Exploration

As proof-of-concept for the utility of these data, we used the MOG platform (v1.8.1) (Singh et al., 2020) to explore transcript co-expression and make functional inferences. We first created a MOG project *S.cerevisiae*_RNA-seq_3457_27.mog. This MOG project combines: (1) the levels of expression of each gene and ORF in the SGD+ORF dataset across 3,457 conditions; (2) gene and ORF metadata; and (3) sample metadata. For each gene and ORF, metadata include: functional annotations (from SGD); MCL cluster memberships with GO enrichment analysis; mean expression levels for RNA-Seq and ribosomal profiling; ribosomal binding evidence; genome location relative to UTRs and CDSs; GC content; length; genomic positional coordinates, orientation; and phylostratal assignment. Sample and study metadata (retrieved from NCBI-SRA)in the MOG project include: study ID, title, summary, reference, design description, library construction protocol, sequencing apparatus; sample title, experimental attributes, number of replicates; replicate name, sequencing depth, base coverage.

To explore the genes regulated by specific conditions, we did differential analysis in MOG, using the Mann–Whitney *U* test for differential expression analysis, with adjusted *p*-values by Benjamini and Hochberg. We chose all genes and ORFs with expression in the control samples or specific stresses [UV mutagenesis (Huang et al., 2017); under 37°C at least 30 min (Andrie et al., 2014; Gupta et al., 2014; Presnyak et al., 2015; Wery et al., 2016; Uwimana et al., 2017)]. We designate genes and ORFs with log fold change > 1 and adjusted *p*-value < 0.01 as upregulated by the stress,” log fold change<−1 and adjust *p*-value < 0.01 as downregulated by the stress.

## Results and Discussion

### Identifying Potential Cryptic Orphan Genes in *Saccharomyces cerevisiae*

*Saccharomyces cerevisiae* has the most extensively sequenced and annotated genome within the *Saccharomyces* genus, or perhaps across eukaryotes. However, despite the large body of research on *S. cerevisiae*, this genome expresses many transcripts not annotated as genes (Carvunis et al., 2012; Pelechano et al., 2013; Smith et al., 2014; Lu et al., 2017; Wu and Knudson, 2018; Blevins et al., 2021), some of *de novo* origin (Carvunis et al., 2012; Vakirlis et al., 2018, 2020; Arendsee et al., 2019a; Van Oss and Carvunis, 2019), some supported with translational evidence (Van Oss and Carvunis, 2019; Blevins et al., 2021). Our overall goal was to generate a comprehensive overview of expression of ORFs, and make these data available in a format that can be readily explored. For this study, we classified all unannotated ORFs (>150 nt) and annotated genes in the *S. cerevisiae* genome according to phylostrata, transcription and translation evidence, and genomic context. We also included yeast ORFs < 150 nt with transcription and/or translation evidence that had been characterized in two previous publications: smORFs (Carvunis et al., 2012) and txORFs (Lu et al., 2017). Figure 1B defines our process and lists the numbers of genes and ORFs identified at each step.

We inferred the oldest phylostratum (PS; Šestak and Domazet-Lošo, 2015) to which each *S. cerevisiae* protein (or candidate protein) could be traced, using the reproducible and customizable *phylostratr* package (Arendsee et al., 2019b; Supplementary Figure 1). Similarity to proteins of cellular organisms (i.e., proteins tracing back to prokaryotes) was designated as PS = 1; no similarity to any protein outside of *S. cerevisiae* was designated as PS = 15 (see Supplementary Material, *S.cerevisiae*_RNA-seq_3457_27.mog for PS assignments for each annotated and unannotated transcript). This analysis infers that fewer than 4% of annotated genes are orphans. In contrast, 54% of unannotated ORFs are orphans (“orphan-ORFs”), 40% are genus-specific (PS = 10–14), and only 6% are more highly conserved (PS = 1–9; Figure 1B).

In fungi, plants, and animals, the mean lengths of CDSs of annotated genes increase during evolution, with CDSs of orphan genes being the shortest (Toll-Riera et al., 2009; Arendsee et al., 2014, 2019a; Palmieri et al., 2014; Van Oss and Carvunis, 2019; Supplementary Figure 8A). The ORFs of yeast also follow a similar trend: average lengths of orphan-ORFs are shorter and average length of ORFs increases with increasing phylostrata (Supplementary Figure 7). Consistent with the finding of Basile (Basile and Elofsson, 2017), the mean GC content for annotated orphan genes in *S. cerevisiae* is slightly lower (though not statistically significant) than that of more conserved genes. Similar to this GC content difference for annotated orphan genes, the Q3-transcribed orphan-ORFs, have a slightly lower mean GC content than the Q3-transcribed ORFs of other phylostratum levels (Supplementary Figure 8B). Vakirlis et al. (2018) reported a higher mean GC content among those orphan genes that have a confirmed *de novo* origin.

### Transcriptional Landscape of Genes and ORFs

Expression of annotated orphan genes is often developmentally localized, up-regulated under environmental stress, or associated with species-specific traits (Guo et al., 2007; Li et al., 2009; Colbourne et al., 2011; Arendsee et al., 2014; Bhandary et al., 2018). For example, more yeast orphans are ribosomally bound under starvation conditions than control conditions (Wilson and Masel, 2011; Carvunis et al., 2012).

We anticipated that a characteristic of many of the orphan-ORFs that are actual genes that have escaped annotation would be sparse-expression. We aimed to identify RNA-Seq samples comprising diverse developmental, genetic, and environmental conditions, to help to capture expressed but unannotated transcripts. To gather RNA-Seq data from as diverse conditions as were available, we collected raw sequence reads and metadata of 3,457 RNA-Seq samples from 177 studies in NCBI-SRA (see *S.cerevisiae*_RNA-seq_3457_27.mog for metadata and counts). The experimental variables across these samples include a variety of mutants, chemical treatments, stresses, and growth stages. We quantified the expression of all 29,354 ORFs and 6,692 annotated genes of *S. cerevisiae* across the 3,457 RNA-Seq samples.

Using RNA-Seq samples drawn from a wide range of conditions has an additional benefit. Functional inference is a particular challenge for orphan genes, which have no homologs in other species, and rarely have recognizable functional domains (Arendsee et al., 2014). Because genes with similar patterns of expression are likely to encode proteins involved in common processes, using datasets incorporating the diverse conditions under which orphans-ORFs or orphan genes might be expressed provides a powerful approach to determine the conditions that induce their expression, and to infer function based on the co-expressed genes of known function.

**FIGURE 2:**
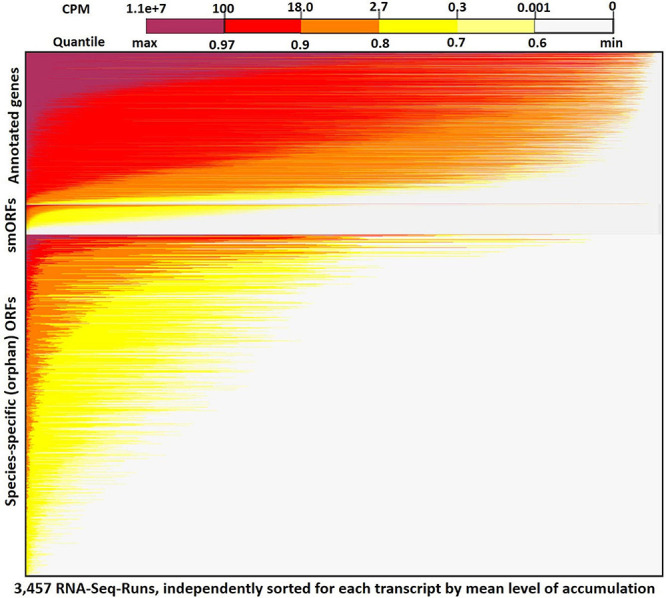
RNA-Seq expression heatmap across 3,457 samples for orphan-ORFs and annotated genes. Top panel, annotated genes (6,692); middle panel, small translated sequences (smORFs) (Carvunis et al., 2012) (1,139); bottom panel, orphan-ORFs (15,805) (see Supplementary Figure 9 for full results). Each row represents a transcript. Within a panel, each transcript is ordered by its mean cpm. Within each row, the 3,457 samples are sorted independently by highest expression of the transcript. The restricted conditions of expression of many orphan-ORFs is visually apparent.

Figure 2 shows a heatmap for expression of all annotated genes, smORFs [sequences encoding small orphan proteins with ribosomal evidence of translation (Carvunis et al., 2012)], and all transcribed orphan-ORFs (>150 nt) across the 3,457 RNA-Seq samples (see Supplementary Figure 9 for heatmap expression plot of all genes and ORFs). The mean expression across all samples for annotated genes is 38 cpm, whereas the mean expression for the Q3-transcribed ORFs is 18 cpm (Supplementary Table 4). Many SGD-annotated genes are expressed in most of the samples. In contrast, as we anticipated based on the erratic pattern of expression of annotated orphan genes, most of the orphan-ORFs show very low expression in most RNA-Seq samples, but accumulate more highly in a few samples. This sporadic expression contributes to the observed lower mean expression of the orphans across all samples. It also demonstrates how transcribed sequences might be missed if smaller, less diverse datasets are considered.

**FIGURE 3:**
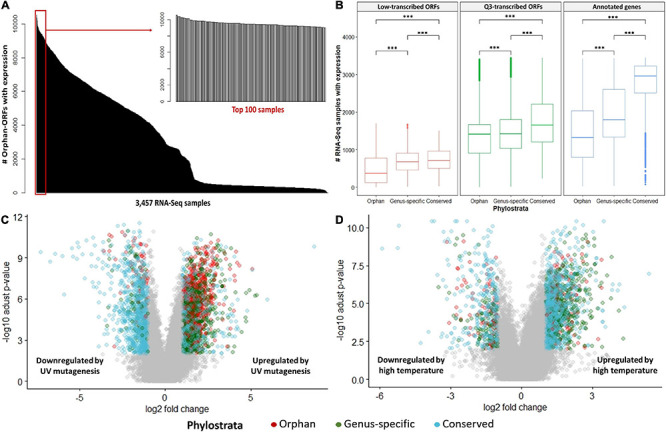
RNA-Seq expression by conditions. **(A)** Number of orphan- ORFs with expression evidence in each RNA-Seq sample. The black bars show distribution of the counts of the 15,809 orphan-ORFs. *X*-axis, 3,457 RNA-Seq samples, sorted by counts. The gray bar inset details the top 100 RNA-Seq samples with the largest number of orphan-ORFs. **(B)** Number of RNA-Seq samples in which gene/ORF is expressed. ^∗∗∗^, *p*-value < 0.001 according to *t*-test. **(C,D)** Volcano plot for differential analysis under UV mutagenesis and high temperature. *X*-axis, log2 fold change of mean expression under control and stress conditions. *Y*-axis, -log10 of adjusted *p*-value in Mann–Whitney *U* test. Differentially expressed genes/ORFs are colored according to phylostrata, and gray points indicates unregulated by stress.

Ninety-nine percent of the 3,457 RNA-Seq samples have transcription evidence for at least one of the orphan-ORFs (Figure 3A). Some samples are particularly rich in orphan-ORFs. For example, 1,000 samples have transcription evidence for >9,000 of the orphan ORFs; these samples grown under conditions of nutritional or chemical stress and studies from different mutant. The phylostrata of transcript (orphan, genus-specific and conserved) and transcript status (low-transcribed ORFs, Q3-transcribed ORFs, and annotated) showed significant effect on the number of RNA-Seq sample with expression for each gene/ORF according to the two-way ANOVA test (Table 1, Figure 3B). Younger genes often expressed in less samples, which is consistent with previous studies. Unannotated ORFs also expressed in less RNA-Seq samples than annotated genes regardless of the phylostrata, that’s one reason why they were omitted from annotation. We chose two stress with sufficient RNA-Seq samples in our dataset as example to verify whether young genes are regulated by stress. Over 2,000 orphan and genus-specific genes and ORFs are upregulated by the UV mutagenesis (Figure 3C and Table 2), and about 1,000 orphan and genus-specific genes and ORFs are upregulated by the high temperature (under 37°C at least 30 min) (Figure 3D and Table 2).

**TABLE 1:** Two-way ANOVA test for comparison of the number of RNA-Seq samples with expression in different transcripts phylostrata and status.

**Source of variation**	**Degrees of freedom**	**Sums of squares**	**Mean squares**	***F* value**	**Pr(>*F*)**
Phylostrata	2	1.63E+10	8.15E+09	31721.7	<2e-16 ***
Status	2	7.33E+09	3.66E+09	14278.5	<2e-16 ***
Phylostrata:Status	4	3.68E+08	9.20E+07	358.1	<2e-16 ***
Residuals	36037	9.26E+10	2.57E+05		

Phylostrata, orphan, genus-specific, and conserved; Status, low-transcribed ORFs, Q3-transcribed ORFs and annotated genes; ***p-value < 0.001.

**TABLE 2:** Numbers of genes/ORFs regulated by UV mutagenesis and temperature stresses.

**Stress**	**Regulation**	**Orphan**	**Genus-specific**	**Conserved**
UV mutagenesis	Upregulated by stress	1,014	1,002	379
	Downregulated by stress	94	147	877
Temperature	Upregulated by stress	272	738	791
	Downregulated by stress	115	120	305

*Mann–Whitney *U* test for differential expression, adjusted *p*-value < 0.01. Log fold change > 1 upregulated by stress, log fold change <−1 downregulated by stress.*

**FIGURE 4:**
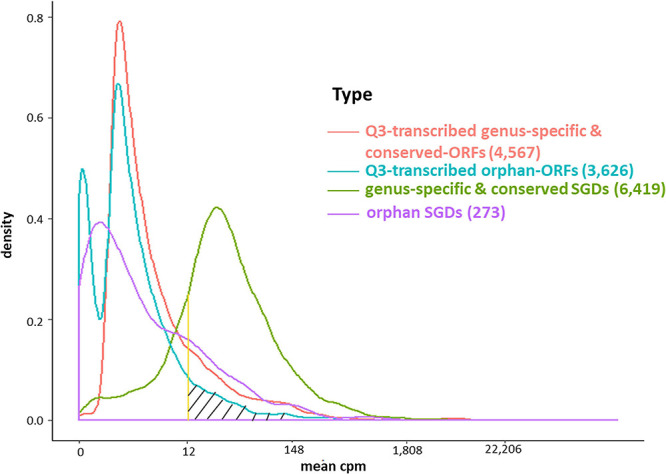
Density plot of mean expression level of transcripts across 3,457 samples for annotated genes and Q3-transcribed ORFs. *X*-axis, *edgeR*-normalized mean expression of genes and ORFs. *Y*-axis, number of transcripts. The area under the curve of the density function represents the probability of a range of mean cpm. The bimodal curve of all orphan-ORFs is attributable to the low mean expression of the smORFs (see Supplementary Figures 10, 11). About half of the Q3-transcribed orphan-ORFs have higher mean expression than annotated orpahn genes. Over 600 orphan-ORFs have a higher mean expression than 10% of annotated conserved genes; 289 orphan-ORFs (gray hatched area) have a higher mean expression than 25% of annotated conserved genes; and, 36 orphan-ORFs have a mean expression higher than 90% of annotated conserved genes (see also Supplementary Table 4).

The conserved SGD-annotated genes have higher mean expression than either the orphan annotated genes, the Q3-transcribed orphan-ORFs, or the Q3-transcribed conserved-ORFs (Kolmogorov–Smirnov Test, *p*-values < 0.001; Figure 4). However, despite their generally sparse distribution, over 600 orphan-ORFs have a higher mean expression than 10% of conserved annotated genes, 289 orphan-ORFs have a mean expression higher than 25% of the conserved annotated genes, and 36 orphan-ORFs have a mean expression higher than 90% of conserved annotated genes (Figure 4 and Supplementary Table 4A).

### Translation Evidence of Genes and ORFs

**FIGURE 5:**
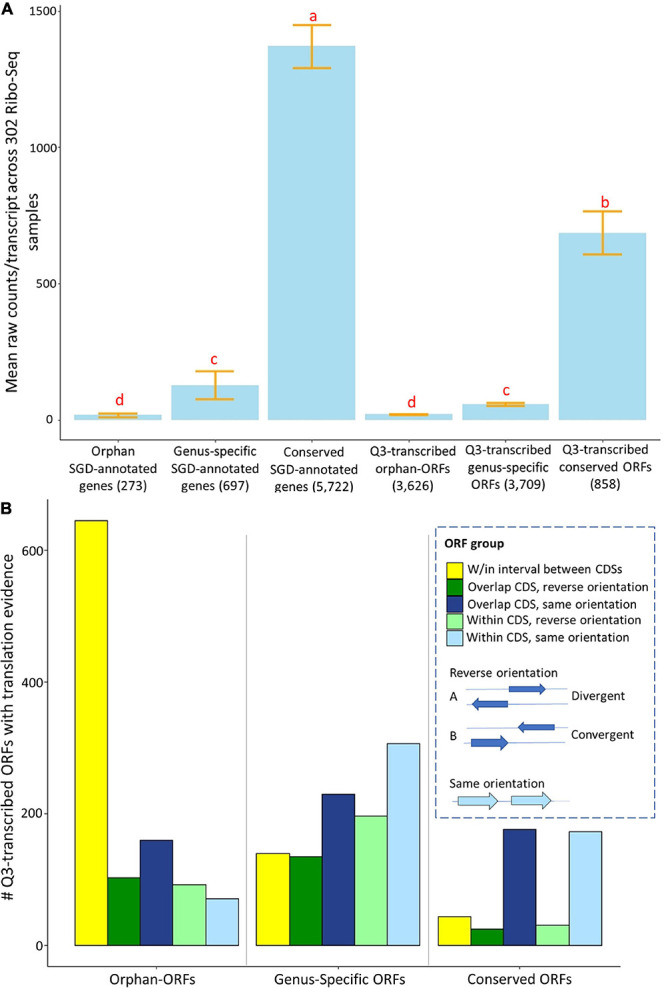
Mean expression and numbers of genes and ORFs with translational evidence, partitioned by phylostrata and genomic context. Ribo-Seq data were analyzed for genes and ORFs across 302 samples using *ribotricer* (Choudhary et al., 2019). **(A)** Mean raw Ribo-Seq counts per transcript for all genes and ORFs. *X*-axis, genes and ORFs as classified by phylostrata. *Y*-axis, mean value of raw read counts. The letters above each bar indicate significance in each group according to a *t*-test (*p*-value cutoff is 0.01). Similar to mean RNA-Seq counts, the conserved genes and conserved-ORFs have more total mean Ribo-Seq counts. **(B)**. The 3,857 Q3-transcribed ORFs that had Ribo-Seq translation evidence were divided into groups according to their relationship to annotated, and the numbers of genes and ORFs with translational evidence was determined. The gene/ORF with mean counts across 302 Ribo-Seq samples higher than 1.83 was consider to have translation evidence. *X*-axis, groups of genes and ORFs, classified by phylostrata. *Y*-axis, number of ORFs in each group. The proportions of ORFs are significantly different among three phylostratal groups according to a chi-square test (*p*-value < 0.001). Over 60% the orphan-ORFs with translation evidence are located in the interval between protein coding sequences (CDSs).

Many RNAs in fungi and animals that have been annotated as “lncRNAs” are associated with ribosomes, and/or have proteomics evidence, indicating some of them may function as protein-coding genes (Wilson and Masel, 2011; Wu et al., 2011; Hangauer et al., 2013; Ruiz-Orera et al., 2015, 2018). To examine translation evidence in our study, we globally evaluated translation evidence, mapping raw reads from 302 ribosomal profiling RNA-Seq (Ribo-Seq) samples in SRA to the unannotated ORFs and annotated genes of *S. cerevisiae* (see Supplementary Material Ribo-Seq_counts.csv and Ribo-Seq_metadata.xlsx for raw counts and metadata). These 302 Ribo-Seq studies include a variety of conditions, but are lacking in representation of many stress conditions. About 52% of Q3-transcribed conserved-ORFs, 27% of genus-specific-ORFs, and 30% of orphan-ORFs have translational evidence among these somewhat limited Ribo-Seq samples (Figure 1B). This compares to 96% of the conserved annotated genes, 27% of genus-specific annotated genes, and 20% of orphan annotated genes. The mean Ribo-Seq raw counts were significantly different (*t*-test *p*-value < 0.001) among classes of transcripts, depending on whether they were orphan, genus-specific, or conserved (Figure 5A). The mean Ribo-Seq raw counts for the low-transcribed ORFs are significantly lower than for the Q3-transcribed ORFs, and the mean Ribo-Seq raw counts for the ORFs with no transcription evidence are 0 or near 0 (Supplementary Figure 13).

The proportions of Q3-transcribed ORFs with translation evidence located within, overlapping, or between annotated CDSs are significantly different among orphan-ORFs, genus-specific-ORFs, and conserved-ORFs (Chi-square test, *p*-value < 0.001) (Figure 5B). Notably, 60% of Q3-transcribed orphan-ORFs with translation evidence are located in the intervals between annotated CDSs, compared to only 14% of the genus-specific ORFs and 10% of the conserved ORFs (Figure 5B).

Since yeast was the first model eukaryotic genome, and has been reannotated over time, it would be expected that most conserved genes are already annotated. However, some genus-specific-genes might have been missed because homology is a major criterion used for genome annotation. Orphan genes, which have no homologs in other species, sparser expression, and likely fewer canonical features (Li et al., 2021), are yet less likely to have been annotated. In total, 1,007 Q3-transcribed genus-specific-ORFs and 1,070 Q3-transcribed orphan-ORFs have ribosomal binding evidence. These transcribed, translated ORFs are candidates for protein-coding genes.

Four hundred and forty-nine of the 858 Q3-transcribed conserved-ORFs also have translation evidence. There are several possible explanations for why a transcript with translation evidence and homologs in other species are not annotated as genes. Some of these conserved-ORFs may be pseudogenes that retain some homology and expression, but have lost functional capacity. Other conserved-ORFs might encode active proteins, by because they are expressed only under limited conditions they might not have been sampled when SGD annotations were made. Still other conserved-ORFs may have been ignored because their ORF codes for a shorter protein than the canonical gene family member. [On average, a Q3-transcribed conserved-ORF is significantly shorter than the homologous annotated gene (*t*-test, *p*-value < 0.001)]. However, it not a given that because an ORF encodes a shorter protein it is non-functional. Translation of a short conserved-ORF might play a regulatory in that it limits translation of a nearby active protein (Wu et al., 2019). Also, shorter homologs of proteins with known function may play a biological role in regulating signal transduction, modulating enzyme activity, and/or affecting protein complexes, potentially competing with their “full-length” homolog (Frith et al., 2006; Storz et al., 2014).

### Network Inference and Co-expression Analysis

To analyze the expression patterns of the ORFs in the context of annotated genes, we optimized correlation and network parameters for the RNA-Seq expression data (Supplementary Section 4), focusing our subsequent interactive co-expression analysis and visualization on a dataset (“SGD + ORF” dataset) composed of 14,885 transcripts (all annotated genes; the 7,054 Q3-transcribed ORFs; and all 1,139 smORFs) across 3,457 RNA-Seq samples.

We then computed the PCC matrix for the SGD+ORF dataset, and partitioned the resultant network with PCC > 0.6 (only consider positive correlation) by Markov chain graph clustering (MCL; van Dongen, 2000) into 544 clusters (Supplementary Table 1 for overview; genes and ORFs with cluster designations at Supplementary Material *S.cerevisiae*_RNA-seq_3457_27.mog). Forty-six percent of the 273 annotated orphan genes and 59% of the 3,899 Q3-transcribed orphan-ORFs are members of clusters containing more than five genes and include genes of known function, thus providing potential for functional inference.

**FIGURE 6:**
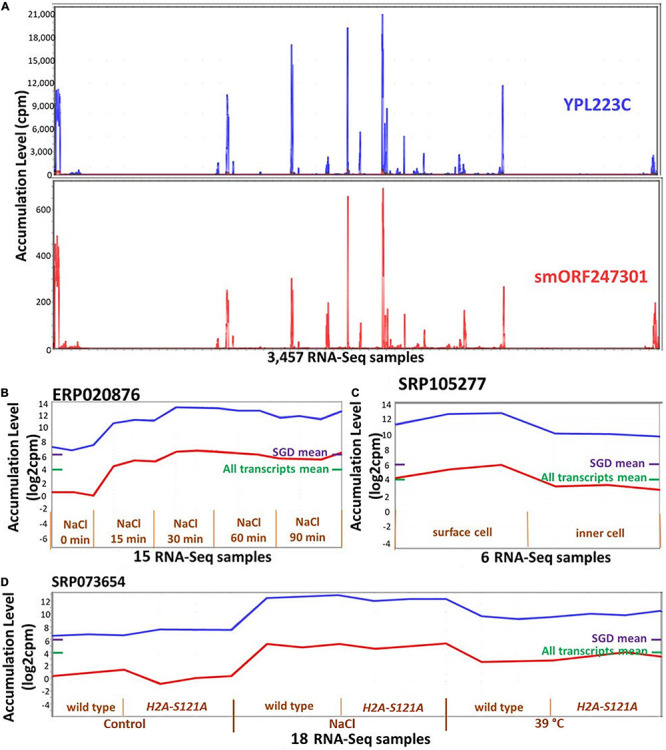
Expression patterns of smORF247301 and YPL223C. *smORF247301* and *YPL223C* are located on adjacent regions of chromosome 16 and are transcribed in convergent orientation. **(A)** Expression patterns are similar (Pearson correlation, 0.95) across 3,457 samples. **(B–D)** Expression patterns for smORF247301 and YPL223C in three studies. *X*-axis, 3 samples per treatment. Purple bar on right side of panels, mean expression level of all annotated genes; Green bar on right side of panels, mean expression level of all annotated genes and all ORFs. **(B)** Expression in response to osmotic stress; **(C)** expression in response to desiccation stress (surface cell under stress); **(D)** expression in response to osmotic and high temperature stress (wild strain *vs H2A-S121A* mutant). Visualizations and co-expression calculations used MetaOmGraph (MOG) (Singh et al., 2020).

It was possible that ORF expression might be correlated with that of adjacent or overlapping annotated genes, i.e., that ORFs are expressed due to a physical proximity to transcribed annotated genes. We used two approaches to evaluate the extent to which such “piggybacking” might occur. In the first approach, we focused on the 390 ORFs that are located completely within UTRs of annotated genes (88% are orphan-ORFs). About 80% of these ORFs have a PCC less than 0.6 (0.6 is the correlation cut-off we used for MCL) with the encompassing annotated genes, however, about 2% (eight) ORFs have a correlation higher than 0.9. In the second approach, we calculated how many ORFs are in the same cluster as nearby annotated genes. To do this, we randomly selected 366 ORFs that were members of clusters, and made test clusters of the same sizes, each cluster containing randomly selected annotated genes and the identical ORFs as in the experimental data. Then, we calculated the distance of each ORF to each annotated gene in the randomly created and the experimental clusters. The distances were not statistically different in the experimental versus the random clusters (*p*-value = 0.16 in a *t*-test for difference). These analysis indicate that the expression of ORFs is not generally associated with their proximity to, or overlap with an annotated gene. However, there is strong support for such a relationship for specific ORFs [e.g., Figure 6, and as reported in Vakirlis et al. (2018)].

About 65% of the Q3-transcribed ORFs could be assigned to clusters in the co-expression matrix. Regardless of whether these ORFs are protein-coding, they could play a biological role. Those with translational activity provide an evidence-based cadre of candidate protein-coding genes with an inferred function based on the genes in the cluster with known functions that could be experimentally tested.

### Alternate Analyses

Here, we used positive pairwise Pearson correlations to infer a network, and MCL to partition this network into clusters; we then determined that these clusters are enriched in genes participating in similar biological processes. However, each combination of network inference and partitioning approaches can supply complementary and different information about potential roles of orphan (and indeed all) genes. Networks could be inferred by, for example, correlation, mutual information (Zhang et al., 2012), or relatedness approaches (Netotea et al., 2014). Pearson correlation is highly sensitive at extracting genes whose expression is linearly correlated across multiple conditions, but misses non-linear co-expression. Weighted correlation approaches may minimize the biological bias due to sample redundancy (Obayashi et al., 2019), but improper cutoff of sample correlation for sample redundancy may lead to lost information in the clustering analysis. Likewise, networks can be partitioned by multiple methods, such as MCL, Modularity (Newman, 2006), and a very promising new approach, Reduced Network Extreme Ensemble Learning (RenEEL; Guo et al., 2019). Each combination of network inference and network partitioning method may provide different strengths and weaknesses in terms of extracting different types of useful biological information. For example, some approaches might be better at identifying signal transduction pathways, others at metabolic pathways, stress-responses, or hub genes and their targets. The large SGD+ORF dataset we provide herein could be analyzed by different approaches. Such analyses would that extract the same relationships would provide support for these relationships; also, comparative analysis would help reveal the strengths and weaknesses of various network inference and partitioning methods for extracting different types of biological information.

We have focused here on unannotated protein-coding transcripts of over 50 aa (except the smORFs, which are smaller); similar investigations could incorporate non-coding RNAs or transcripts encoding very small proteins. The information resulting from such studies could be incorporated into a new MOG project to enable interactive analysis and visualization.

### Gene Ontology Enrichment Analysis for Co-expressed Clusters

**FIGURE 7:**
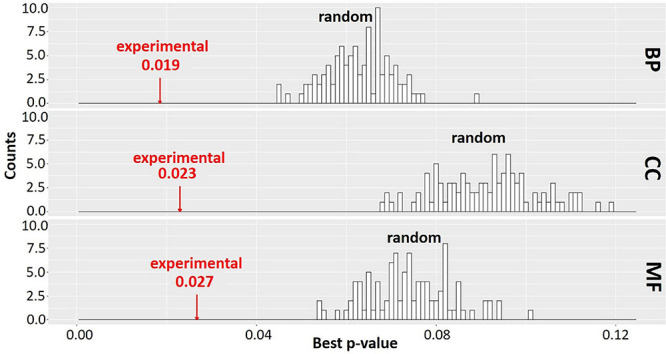
Gene Ontology (GO) enrichment analysis of experimental data and random permutation test distribution. A Pearson correlation matrix of the Saccharomyces Genome Database (SGD)+ORF dataset was partitioned into clusters by Markov Cluster (MCL). Best *p*-values (mean of the lowest adjusted *p*-values for GO terms) were determined across all clusters of the experimental data and all clusters of random permutations, similar to Mentzen and Wurtele (2008); Supplementary Section 4. Red arrow, experimental data. Black bars, best *p*-value of 100 randomly obtained permutations with size and number of clusters identical to experimental data. BP, biological process; CC, cellular component; MF, molecular function. The clustering result is significantly better for experimental data than any random permutation.

In order to evaluate the significance of the clustering results, we compared the extent of enrichment of GO terms in the set of clusters obtained from MCL-partitioning experimental data to that of 100 randomly generated sets of clusters. For each randomly generated set, the number of clusters and the number of genes per cluster were held the same as the set of clusters from the experimental data; however, the genes assigned to each cluster were changed by random permutation. The best adjusted *p*-value for enriched GO terms was recorded for each cluster and averaged across all clusters to obtain a mean best *p*-value (Mentzen and Wurtele, 2008; Figure 7). Distribution of the *p*-values for GO terms in the 100 sets of randomized clusters was compared to that of the experimental data (red arrows in Figure 7). In our study, for each GO ontology category BP, CC, and MF, the best mean *p*-values for the experimental data are 0.019, 0.023, and 0.027, respectively. These values are significantly better than those of any of the randomly obtained cluster sets, indicating that the MCL gene clusters derived from the experimental data is not random. Most co-expression clusters are composed of genes and ORFs distributed across spatially diverse regions of the genome. Co-expressed genes are implicated as being involved in a similar biological process (Eisen et al., 1998; Spellman et al., 1998). This study is based on over 3,000 RNA-Seq samples further strengthens the likelihood that genes in each cluster might share a related biological process. All genes and ORFs, as partitioned into clusters by MCL, are provided in Supplementary Material *S.cerevisiae*_RNA-seq_3457_27.mog.

### Exploring Gene Function: Case Study, Cluster 112

**FIGURE 8:**
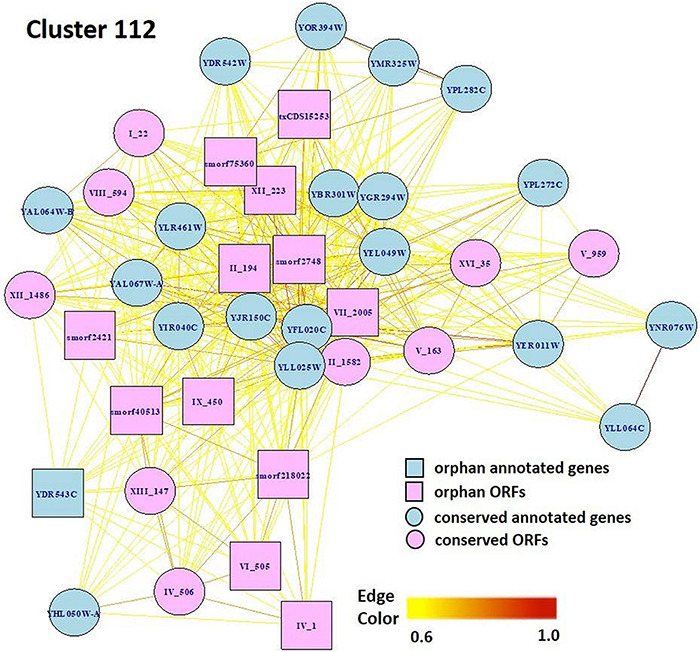
Network view of genes and ORFs in Cluster 112, a cluster enriched in seripauperins and other stress-responsive genes. A Pearson correlation matrix of the SGD+ORF dataset was partitioned into clusters by MCL. Edge colors, Pearson correlations(0.6–1.0). Visualization by *igraph* in R (Csárdi and Nepusz, 2006).

**FIGURE 9:**
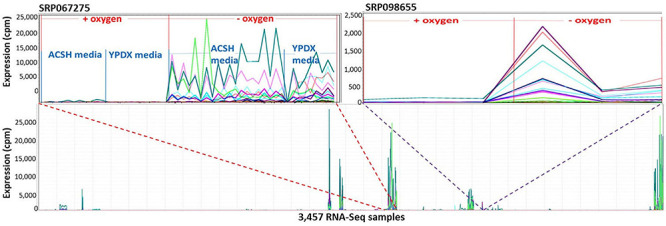
The 41 genes and ORFs in Cluster 112 respond to anoxia. A Pearson correlation matrix of the SGD+ORF dataset was partitioned into clusters by MCL. The 41 genes and ORFs in Cluster 112 are co-expressed across multiple conditions. *X*-axis, 3,457-samples, sorted by study. *Y*-axis, expression values. Each line represents the expression pattern of a single gene or ORF. Top left inset, zoomed-in to visualize Study SRP067275. RNA-Seq samples sorted by: aerobic or anaerobic condition, media composition, and growth phase. ACSH, Ammonia Fiber Expansion-(AFEX-) pretreated corn stover hydrolysate. YPDX, YP media containing 60 and 30 g/L xylose. The genes and ORFs are up-regulated in response to anoxia, regardless of changes in growth media. Top right inset, zoom-in to visualize Study SRP098655. The genes and ORFs are up-regulated in response to anoxia, regardless of changes in growth media. No ORF in Cluster 112 is located near an annotated gene in Cluster 112. Visualizations and co-expression calculations by MOG (Singh et al., 2020).

Markov Cluster Cluster 112 (Figure 8) contains 20 annotated genes and 21 unannotated ORFs dispersed over 14 chromosomes. Twelve of the genes are in the seripauperin (*PAU*) family. *PAU*-rich co-expressed gene clusters have also been identified in independent microarray studies (Magwene and Kim, 2004; Orellana et al., 2014). The precise molecular function of the *PAU* genes is not known. However, many *PAU*s are induced by low temperature and anaerobic conditions, and repressed by heme (Rachidi et al., 2000) and individual *PAU* proteins confer resistances to biotic and abiotic stresses (Rivero et al., 2015). *YER011W* and *YJR150C*, both cell wall mannoproteins are also in Cluster 112, are localized to the same cellular compartments as *PAU*s and are also induced under anaerobic conditions (Kowalski et al., 1995; Kitagaki et al., 1997; Sertil et al., 1997; Cohen et al., 2001). The other annotated genes in this cluster have no functional description. GO enrichment analysis identified eight GO terms as significantly over-represented in Cluster 112 (Table 3). Figure 9 represents a case study of an approach to develop a meaningful hypothesis. The example shows the co-expression of the genes and ORFs in Cluster 112 across all 3457 samples of the RNA-Seq SGD+ORF dataset (lower panel). Two studies that evaluate oxygen content as an experimental variable are highlighted. Study SRP067275 compares four growth stages of the stress-tolerant yeast strain GLBRCY22-3 grown in YPDX and ACSH media, with and without oxygen (McIlwain et al., 2016; Figure 9, upper left); the expression of the genes and ORFs in Cluster 112 is higher under anaerobic conditions, irrespective of media or growth stage. Study SRP098655 compares *OLE1*-repressible strains growing under anaerobic and aerobic conditions (Degreif et al., 2017; Figure 9, upper right); expression of genes and ORFs in Cluster 112 is induced in cells grown under anaerobic conditions. These expression patterns indicate the genes and the ORFs in this cluster might be sensitive to anoxia, and might play a role in cellular response to this stress.

**TABLE 3:** Significantly enriched GO terms in Cluster 112.

**Ontology**	**GO name**	**Adjust *p*-value**
MF	Structural constituent of cell wall	1.90E-27
BP	Response to stress	3.51E-27
CC	Fungal-type cell wall	1.62E-20
BP	Fungal-type cell wall organization	1.34E-19
CC	Fungal-type vacuole	3.60E-05
CC	Cell wall	1.99E-03
CC	Extracellular region	6.18E-03
CC	Anchored component of membrane	1.87E-02

*Based on the GO terms assigned to the gene members of known function, Cluster 112 is enriched in the GO terms shown in Table 3. The results indicate a possible role in stress response related to the cell wall for the ORF members of Cluster 112 (see *S. cerevisiae*_RNA- seq_3457_27.mog for complete clustering and ontology results).*

### Exploring Gene Function: Case Study, smORF247301

Though rare, some transcribed ORFs that are located near or in an existing gene share a similar transcription pattern. An example is *smORF247301*, one of the most highly expressed smORFs, which is 77 nt upstream of *YPL223C* (Figure 6). MOG analysis indicates *smORF247301* and the nearby annotated gene *YPL223C* have a PCC of 0.95 across the 3,457 RNA-Seq samples. *smORF247301* is located on the “+” strand of chromosome 16, while *YPL223C* is on the “−” strand of the same chromosome. The CDS of *YPL223C* is 507 nt, while *smORF247301* is 33 nt. *YPL223C* is more highly expressed than *smORF247301*. *YPL223C*, a hydrophilin gene that is essential in surviving desiccation-rehydration, is regulated by the high-osmolarity glycerol (HOG) pathway (Garay-Arroyo et al., 2000), and induced by osmotic, ionic, oxidative, heat shock and heavy metals stresses. Analysis using MOG shows *smORF247301* and *YPL223C* have increased expression in response to osmotic, heat, and desiccation stresses in three independent studies (Figures 6B–D). *smORF247301* has translation evidence [(Carvunis et al., 2012) and this study].

It is possible that the transcription and translation of *smORF247301* is “noise” (Eling et al., 2019) associated with the expression of the nearby *YPL223C*. A second possibility is that *smORF247301* is a young, not-yet-annotated gene. It might be “piggybacking” on the expression apparatus of *YPL223C*. However, *smORF247301* and *YPL223C* are transcribed in a *convergent* orientation (Figure 5B); thus, the process, described by Vakirlis et al. (2018), whereby two transcripts in divergent orientation (Figure 5B) are co-expressed via a common bidirectional promoter would not apply in the case of *smORF247301* and *YPL223C*. A different “piggybacking” mechanism might apply: perhaps, due to its location in open chromatin, *smORF247301* is provided with a ready-made exposure to transcription factors when gene *YPL223C* is transcribed. If a transcript (e.g., *smORF247301*) conferred a survival advantage under the same conditions as did its established neighboring gene (e.g., *YPL223C*), it could emerge as a new, co-expressed, gene by this mechanism.

Five hundred and thirty-seven orphan-ORFs with transcription and translation evidence are in physical proximity to an annotated gene and are transcribed in a divergent orientation (see Supplementary Material, divergent_pairs.csv). Of these pairs, 12 are co-expressed (PCC > 0.6); these 12 ORFs are potentially co-expressed by a bidirectional promoter [e.g., as described by Vakirlis et al. (2018)] The 525 orphan-ORFs that are not co-expressed, might still be controlled by a bidirectional promoter, because yeast ORFs can be transcribed by a bidirectional promoter, but not be correlated in expression because they are influenced by different transcription factors (Xu et al., 2009).

## Conclusion

In this study we have globally assessed the accumulation of transcripts representing 36,046 annotated genes and unannotated ORFs of *S. cerevisiae* across 3,457 public RNA-Seq samples derived from diverse biological conditions. Ninety-five percent of the transcribed ORFs are orphans or genus-specific. Despite a strong tendency to be transcribed only under restricted conditions, 269 orphan-ORFs had mean levels of transcription across all conditions greater than 25% of annotated genes. Over 1,600 transcribed ORFs with translation evidence are members of co-expression clusters, providing additional clues as to a potential function.

The proportion of transcribed and translated ORFs that are functional genes is unknown. The SGD+ORF dataset assembled herein represents expression of annotated genes and unannotated ORFs under multiple conditions; it is delivered in a readily explorable, user-friendly format via the MOG platform. Combining this network-informed view of aggregate RNA-Seq data with text-mining of sample and gene metadata provides a powerful approach to develop novel, experimentally testable hypotheses on the potential functions of as-yet-unannotated transcripts.

## Data Availability Statement

The datasets presented in this study can be found in online repositories. The names of the repository/repositories and accession number(s) can be found in the article/ Supplementary Material.

## Author Contributions

JL and EW conceived of the project and drafted the manuscript. JL carried out the design of the study and performed the statistical analysis. US participated in the visualization on MOG and provided Ribo-Seq analysis code and guidance. ZA contributed to the phylostrata analysis. All authors contributed, read, and approved the final manuscript.

## Conflict of Interest

The authors declare that the research was conducted in the absence of any commercial or financial relationships that could be construed as a potential conflict of interest.

## Publisher’s Note

All claims expressed in this article are solely those of the authors and do not necessarily represent those of their affiliated organizations, or those of the publisher, the editors and the reviewers. Any product that may be evaluated in this article, or claim that may be made by its manufacturer, is not guaranteed or endorsed by the publisher.

## Footnotes

1
https://github.com/urmi-21/MetaOmGraph

2
https://datahub.io/lijing28101/yeast_supplementary

3
https://github.com/lijing28101/SPARK_MCL

## Abbreviations

CDS: protein coding sequence
ORF: open reading frame
MOG: MetaOmGraph
SGD: Saccharomyces Genome Database
Q3-transcribed ORF: ORFs with mean expression values in the upper quantile of mean expression
PCC: Pearson Correlation Coefficient
MCL: Markov Cluster
GO: Gene Ontology
NCBI-SRA: The National Center for Biotechnology Information-Sequence Read Archive
BP: biological process
CC: cellular component
MF: molecular function.

## References

[B1] Andrews S. J. Rothnagel J. A. (2014). Emerging evidence for functional peptides encoded by short open reading frames. *Nat. Rev. Genet.* 15 193–204. 10.1038/nrg3520 24514441

[B2] Andrie J. M. Wakefield J. Akey J. M. (2014). Heritable variation of mRNA decay rates in yeast. *Genome Res.* 24 2000–2010. 10.1101/gr.175802.114 25258386 PMC4248316

[B3] Arendsee Z. Li J. Singh U. Bhandary P. Seetharam A. Wurtele E. S. (2019a). Fagin: synteny-based phylostratigraphy and finer classification of young genes. *BMC Bioinformatics* 20:440. 10.1186/s12859-019-3023-y 31455236 PMC6712868

[B4] Arendsee Z. Li J. Singh U. Seetharam A. Dorman K. Wurtele E. S. (2019b). Phylostratr: a framework for phylostratigraphy. *Bioinformatics* 35 3617–3627. 10.1093/bioinformatics/btz171 30873536

[B5] Arendsee Z. W. Li L. Wurtele E. S. (2014). Coming of age: orphan genes in plants. *Trends Plant Sci.* 19 698–708. 10.1016/j.tplants.2014.07.003 25151064

[B6] Bao Z. Clancy M. A. Carvalho R. F. Elliott K. Folta K. M. (2017). Identification of novel growth regulators in plant populations expressing random peptides. *Plant Physiol.* 175 619–627. 10.1104/pp.17.00577 28807931 PMC5619883

[B7] Barroso G. V. Puzovic N. Dutheil J. Y. (2018). The evolution of gene-specific transcriptional noise is driven by selection at the pathway level. *Genetics* 208 173–189. 10.1534/genetics.117.300467 29097405 PMC5753856

[B8] Basile W. Elofsson A. (2017). The number of orphans in yeast and fly is drastically reduced by using combining searches in both proteomes and genomes. *BioRxiv* [Preprint]. 10.1101/185983

[B9] Berardini T. Z. Reiser L. Li D. Mezheritsky Y. Muller R. Strait E. (2015). The *Arabidopsis* information resource: making and mining the ‘Gold Standard’ annotated reference plant genome. *Genesis* 53 474–485. 10.1002/dvg.22877 26201819 PMC4545719

[B10] Bhandary P. Seetharam A. S. Arendsee Z. W. Hur M. Wurtele E. S. (2018). Raising orphans from a metadata morass: a researcher’s guide to Re-Use of Public ’omics data. *Plant Sci.* 267 32–47. 10.1016/j.plantsci.2017.10.014 29362097

[B11] Blevins W. R. Ruiz-Orera J. Messeguer X. Blasco-Moreno B. Villanueva-Cañas J. L. Espinar L. (2021). Uncovering de novo gene birth in yeast using deep transcriptomics. *Nat. Commun.* 12:604. 10.1038/s41467-021-20911-3 33504782 PMC7841160

[B12] Bray N. L. Pimentel H. Melsted P. Pachter L. (2016). Near-optimal probabilistic RNA-seq quantification. *Nat. Biotechnol.* 34 525–527. 10.1038/nbt.3519 27043002

[B13] Bushnell B. (2014). *BBMap: A Fast, Accurate, Splice-Aware Aligner. LBNL-7065E.* Berkeley, CA: Lawrence Berkeley National Lab.

[B14] Cantarel B. L. Korf I. Robb S. M. C. Parra G. Ross E. Moore B. (2008). MAKER: an easy-to-use annotation pipeline designed for emerging model organism genomes. *Genome Res.* 18 188–196. 10.1101/gr.6743907 18025269 PMC2134774

[B15] Carvunis A.-R. Rolland T. Wapinski I. Calderwood M. A. Yildirim M. A. Simonis N. (2012). Proto-genes and *de Novo* gene birth. *Nature* 487 370–374. 10.1038/nature11184 22722833 PMC3401362

[B16] Chen S. Krinsky B. H. Long M. (2013). New genes as drivers of phenotypic evolution. *Nat. Rev. Genet.* 14 645–660. 10.1038/nrg3521 23949544 PMC4236023

[B17] Chew G.-L. Pauli A. Rinn J. L. Regev A. Schier A. F. Valen E. (2013). Ribosome profiling reveals resemblance between long non-coding RNAs and 5’ leaders of coding RNAs. *Development* 140 2828–2834. 10.1242/dev.098343 23698349 PMC3678345

[B18] Choudhary S. Li W. Smith A. D. (2019). Accurate detection of short and long active ORFs using Ribo-Seq data. *Bioinformatics* 36 2053–2059. 10.1093/bioinformatics/btz878 31750902 PMC7141849

[B19] Cohen B. D. Sertil O. Abramova N. E. Davies K. J. A. Lowry C. V. (2001). Induction and repression of DAN1 and the family of anaerobic mannoprotein genes in *Saccharomyces cerevisiae* occurs through a complex array of regulatory sites. *Nucleic Acids Res.* 29 799–808. 10.1093/nar/29.3.799 11160904 PMC30382

[B20] Colbourne J. K. Pfrender M. E. Gilbert D. Thomas W. K. Tucker A. Oakley T. H. (2011). The ecoresponsive genome of daphnia pulex. *Science* 331 555–561. 10.1126/science.1197761 21292972 PMC3529199

[B21] Csárdi G. Nepusz T. (2006). *The Igraph Software Package for Complex Network Research.* InterJournal Complex Systems:1695. Available online at: http://igraph.sf.net (accessed February 26, 2020).

[B22] Degreif D. de Rond T. Bertl A. Keasling J. D. Budin I. (2017). Lipid engineering reveals regulatory roles for membrane fluidity in yeast flocculation and oxygen-limited growth. *Metab. Eng.* 41 46–56. 10.1016/j.ymben.2017.03.002 28323063

[B23] Dillies M.-A. Rau A. Aubert J. Hennequet-Antier C. Jeanmougin M. Servant N. (2013). A comprehensive evaluation of normalization methods for illumina high-throughput RNA sequencing data analysis. *Brief. Bioinform.* 14 671–683. 10.1093/bib/bbs046 22988256

[B24] Domazet-Lošo T. Brajković J. Tautz D. (2007). A phylostratigraphy approach to uncover the genomic history of major adaptations in metazoan lineages. *Trends Genet.* 23 533–539. 10.1016/j.tig.2007.08.014 18029048

[B25] Eisen M. B. Spellman P. T. Brown P. O. Botstein D. (1998). Cluster analysis and display of genome-wide expression patterns. *Proc. Natl. Acad. Sci. U.S.A.* 95 14863–14868. 10.1073/pnas.95.25.14863 9843981 PMC24541

[B26] Eling N. Morgan M. D. Marioni J. C. (2019). Challenges in measuring and understanding biological noise. *Nat. Rev. Genet.* 20 536–548. 10.1038/s41576-019-0130-6 31114032 PMC7611518

[B27] ENCODE Project Consortium (2012). An integrated encyclopedia of DNA elements in the human genome. *Nature* 489 57–74. 10.1038/nature11247 22955616 PMC3439153

[B28] Frith M. C. Forrest A. R. Nourbakhsh E. Pang K. C. Kai C. Kawai J. (2006). The abundance of short proteins in the mammalian proteome. *PLoS Genet.* 2:e52. 10.1371/journal.pgen.0020052 16683031 PMC1449894

[B29] Garay-Arroyo A. Colmenero-Flores J. M. Garciarrubio A. Covarrubias A. A. (2000). Highly hydrophilic proteins in prokaryotes and eukaryotes are common during conditions of water deficit. *J. Biol. Chem.* 275 5668–5674. 10.1074/jbc.275.8.5668 10681550

[B30] González C. Lazcano M. Valdés J. Holmes D. S. (2016). Bioinformatic analyses of unique (Orphan) core genes of the genus *Acidithiobacillus*: functional inferences and use as molecular probes for genomic and metagenomic/transcriptomic interrogation. *Front. Microbiol.* 7:2035. 10.3389/fmicb.2016.02035 28082953 PMC5186765

[B31] Grandaubert J. Bhattacharyya A. Stukenbrock E. H. (2015). RNA-Seq-based gene annotation and comparative genomics of four fungal grass pathogens in the genus *Zymoseptoria* identify novel orphan genes and species-specific invasions of transposable elements. *G3* 5 1323–1333. 10.1534/g3.115.017731 25917918 PMC4502367

[B32] Gubala A. M. Schmitz J. F. Kearns M. J. Vinh T. T. Bornberg-Bauer E. Wolfner M. F. (2017). The *Goddard* and *Saturn* genes are essential for *Drosophila* male fertility and may have arisen de novo. *Mol. Biol. Evol.* 34 1066–1082. 10.1093/molbev/msx057 28104747 PMC5400382

[B33] Guo J. Singh P. Bassler K. E. (2019). Reduced network extremal ensemble learning (RenEEL) scheme for community detection in complex networks. *Sci. Rep.* 9:14234. 10.1038/s41598-019-50739-3 31578406 PMC6775136

[B34] Guo W.-J. Li P. Ling J. S-P Ye (2007). Significant comparative characteristics between orphan and nonorphan genes in the rice (*Oryza Sativa* L.). *Genome* 2007:21676. 10.1155/2007/21676 18273382 PMC2216055

[B35] Gupta I. Clauder-Münster S. Klaus B. Järvelin A. I. Aiyar R. S. Benes V. (2014). Alternative polyadenylation diversifies post-transcriptional regulation by selective RNA–protein interactions. *Mol. Syst. Biol.* 10:719. 10.1002/msb.135068 24569168 PMC4023391

[B36] Hangauer M. J. Vaughn I. W. McManus M. T. (2013). Pervasive transcription of the human genome produces thousands of previously unidentified long intergenic noncoding RNAs. *PLoS Genet.* 9:e1003569. 10.1371/journal.pgen.1003569 23818866 PMC3688513

[B37] Hoen D. R. Bureau T. E. (2015). Discovery of novel genes derived from transposable elements using integrative genomic analysis. *Mol. Biol. Evol.* 32 1487–1506. 10.1093/molbev/msv042 25713212

[B38] Hoff K. J. Lange S. Lomsadze A. Borodovsky M. Stanke M. (2016). BRAKER1: unsupervised RNA-Seq-based genome annotation with genemark-ET and AUGUSTUS. *Bioinformatics* 32 767–769. 10.1093/bioinformatics/btv661 26559507 PMC6078167

[B39] Hsu P. Y. Calviello L. Wu H. Y. L. Li F. W. Rothfels C. J. Ohler U. (2016). Super-resolution ribosome profiling reveals unannotated translation events in *Arabidopsis*. *Proc. Natl. Acad. Sci. U.S.A.* 113 E7126–E7135. 10.1073/pnas.1614788113 27791167 PMC5111709

[B40] Huang M. Bao J. Hallström B. M. Petranovic D. Nielsen J. (2017). Efficient protein production by yeast requires global tuning of metabolism. *Nat. Commu.* 8:1131. 10.1038/s41467-017-00999-2 29070809 PMC5656615

[B41] Ji Z. Song R. Regev A. Struhl K. (2015). Many LncRNAs, 5’UTRs, and pseudogenes are translated and some are likely to express functional proteins. *ELife* 4:e08890. 10.7554/eLife.08890 PMC4739776 26687005

[B42] Khalturin K. Hemmrich G. Fraune S. Augustin R. Bosch T. C. G. (2009). More than just orphans: are taxonomically-restricted genes important in evolution? *Trends Genet.* 25 404–413. 10.1016/j.tig.2009.07.006 19716618

[B43] Kim D. Langmead B. Salzberg S. L. (2015). HISAT: a fast spliced aligner with low memory requirements. *Nat. Methods* 12 357–360. 10.1038/nmeth.3317 25751142 PMC4655817

[B44] Kitagaki H. Shimoi H. Itoh K. (1997). Identification and analysis of a static culture-specific cell wall protein, Tir1p/Srp1p in *Saccharomyces cerevisiae*. *Eur. J. Biochem.* 249 343–349. 10.1111/j.1432-1033.1997.t01-1-00343.x 9363789

[B45] Kowalski L. R. Kondo K. Inouye M. (1995). Cold-shock induction of a family of TIP1-related proteins associated with the membrane in *Saccharomyces cerevisiae*. *Mol. Microbiol.* 15 341–353. 10.1111/j.1365-2958.1995.tb02248.x 7746155

[B46] Landry C. R. Zhong X. Nielly-Thibault L. Roucou X. (2015). Found in translation: functions and evolution of a recently discovered alternative proteome. *Curr. Opin. Struct. Biol.* 32 74–80. 10.1016/j.sbi.2015.02.017 25795211

[B47] Li J. Arendsee Z. Singh U. Wurtele E. S. (2020). Landscape of the dark transcriptome revealed through re-mining massive RNA-Seq data. *BioRxiv* [Preprint]. 10.1101/671263 BioRxiv: 671263, PMC8415361 34484307

[B48] Li J. Singh U. Bhandary P. Campbell J. Arendsee Z. Seetharam A. S. (2021). Foster thy young: enhanced prediction of orphan genes in assembled genomes. *BioRxiv* [Preprint]. 10.1101/2019.12.17.880294 PMC9023268 34928390

[B49] Li L. Foster C. M. Gan Q. Nettleton D. James M. G. Myers A. M. (2009). Identification of the novel protein QQS as a component of the starch metabolic network in *Arabidopsis* leaves. *Plant J.* 58 485–498. 10.1111/j.1365-313X.2009.03793.x 19154206

[B50] Lloréns-Rico V. Cano J. Kamminga T. Gil R. Latorre A. Chen W. H. (2016). Bacterial antisense RNAs are mainly the product of transcriptional noise. *Sci. Adv.* 2:e1501363. 10.1126/sciadv.1501363 26973873 PMC4783119

[B51] Lu T. C. Leu J. Y. Lin W. C. (2017). A comprehensive analysis of transcript-supported de novo genes in *Saccharomyces* sensu stricto yeasts. *Mol. Biol. Evol.* 34 2823–2838. 10.1093/molbev/msx210 28981695 PMC5850716

[B52] Magwene P. M. Kim J. (2004). Estimating genomic coexpression networks using first-order conditional independence. *Genome Biol.* 5:R100. 10.1186/gb-2004-5-12-r100 15575966 PMC545795

[B53] McIlwain S. J. Peris D. Sardi M. Moskvin O. V. Zhan F. Myers K. S. (2016). Genome sequence and analysis of a stress-tolerant, wild-derived strain of *Saccharomyces cerevisiae* used in biofuels research. *G3* 6 1757–1766. 10.1534/g3.116.029389 27172212 PMC4889671

[B54] McLysaght A. Hurst L. D. (2016). Open questions in the study of de novo genes: what, how and why. *Nat. Rev. Genet.* 17 567–578. 10.1038/nrg.2016.78 27452112

[B55] Menschaert G. Van Criekinge W. Notelaers T. Koch A. CrappÃ J. Gevaert K. (2013). Deep proteome coverage based on ribosome profiling aids mass spectrometry-based protein and peptide discovery and provides evidence of alternative translation products and near-cognate translation initiation events. *Mol. Cell. Proteomics* 12 1780–1790. 10.1074/mcp.M113.027540 23429522 PMC3708165

[B56] Mentzen W. I. Wurtele E. S. (2008). Regulon organization of *Arabidopsis*. *BMC Plant Biol.* 8:99. 10.1186/1471-2229-8-99 18826618 PMC2567982

[B57] Meyer I. M. Durbin R. (2004). Gene structure conservation aids similarity based gene prediction. *Nucleic Acids Res.* 32 776–783. 10.1093/nar/gkh211 14764925 PMC373336

[B58] Neme R. Amador C. Yildirim B. McConnell E. Tautz D. (2017). Random sequences are an abundant source of bioactive RNAs or peptides. *Nat. Ecol. Evol.* 1:127. 10.1038/s41559-017-0127 28580432 PMC5447804

[B59] Netotea S. Sundell D. Street N. R. Hvidsten T. R. (2014). ComPlEx: conservation and divergence of co-expression networks in *A. thaliana*, *Populus* and *O. sativa*. *BMC Genomics* 15:106. 10.1186/1471-2164-15-106 24498971 PMC3925997

[B60] Newman M. E. J. (2006). Modularity and community structure in networks. *Proc. Natl. Acad. Sci. U.S.A.* 103 8577–8582. 10.1073/pnas.0601602103 16723398 PMC1482622

[B61] Obayashi T. Kagaya Y. Aoki Y. Tadaka S. Kinoshita K. (2019). COXPRESdb v7: a gene coexpression database for 11 animal species supported by 23 coexpression platforms for technical evaluation and evolutionary inference. *Nucleic Acids Res.* 47 D55–D62. 10.1093/nar/gky1155 30462320 PMC6324053

[B62] Olexiouk V. Van Criekinge W. Menschaert G. (2017). An update on SORFs.Org: a repository of small ORFs identified by ribosome profiling. *Nucleic Acids Res.* 46 D497–D502. 10.1093/nar/gkx1130 PMC5753181 29140531

[B63] Orellana M. Aceituno F. F. Slater A. W. Almonacid L. I. Melo F. Agosin E. (2014). Metabolic and transcriptomic response of the wine yeast *Saccharomyces cerevisiae* strain EC1118 after an oxygen impulse under carbon-sufficient, nitrogen-limited fermentative conditions. *FEMS Yeast Res.* 14 412–424. 10.1111/1567-1364.12135 24387769

[B64] Palmieri N. Kosiol C. Schlötterer C. (2014). The life cycle of drosophila orphan genes. *ELife* 3:e01311. 10.7554/eLife.01311 24554240 PMC3927632

[B65] Pelechano V. Wei W. Steinmetz L. M. (2013). Extensive transcriptional heterogeneity revealed by isoform profiling. *Nature* 497 127–131. 10.1038/nature12121 23615609 PMC3705217

[B66] Pertea M. Shumate A. Pertea G. Varabyou A. Chang Y. C. Madugundu A. K. (2018). CHESS: a new human gene catalog curated from thousands of large-scale RNA sequencing experiments reveals extensive transcriptional noise. *Genome Biol.* 19 1–14. 10.1186/s13059-018-1590-2 30486838 PMC6260756

[B67] Prabh N. Rödelsperger C. (2016). Are orphan genes protein-coding, prediction artifacts, or non-coding RNAs? *BMC Bioinformatics* 17:226. 10.1186/s12859-016-1102-x 27245157 PMC4888513

[B68] Presnyak V. Alhusaini N. Chen Y. H. Martin S. Morris N. Kline N. (2015). Codon optimality is a major determinant of mRNA stability. *Cell* 160 1111–1124. 10.1016/j.cell.2015.02.029 25768907 PMC4359748

[B69] Proux-Wéra E. Armisén D. Byrne K. P. Wolfe K. H. (2012). A pipeline for automated annotation of yeast genome sequences by a conserved-synteny approach. *BMC Bioinformatics* 13:237. 10.1186/1471-2105-13-237 22984983 PMC3507789

[B70] Quinlan A. R. Hall I. M. (2010). BEDTools: a flexible suite of utilities for comparing genomic features. *Bioinformatics* 26 841–842. 10.1093/bioinformatics/btq033 20110278 PMC2832824

[B71] Rachidi N. Martinez M. J. Barre P. Blondin B. (2000). *Saccharomyces cerevisiae* PAU genes are induced by Anaerobiosis. *Mol. Microbiol.* 35 1421–1430. 10.1046/j.1365-2958.2000.01807.x 10760143

[B72] Rice P. Longden I. Bleasby A. (2000). EMBOSS: the european molecular biology open software suite. *Trends Genet.* 16 276–277. 10.1016/S0168-9525(00)02024-2 10827456

[B73] Rivero D. Berná L. Stefanini I. Baruffini E. Bergerat A. Csikász-Nagy A. (2015). Hsp12p and PAU genes are involved in ecological interactions between natural yeast strains. *Environ. Microbiol.* 17 3069–3081. 10.1111/1462-2920.12950 26079802

[B74] Robinson M. D. McCarthy D. J. Smyth G. K. (2010). EdgeR: a bioconductor package for differential expression analysis of digital gene expression data. *Bioinformatics* 26 139–140. 10.1093/bioinformatics/btp616 19910308 PMC2796818

[B75] Ruiz-Orera J. Hernandez-Rodriguez J. Chiva C. Sabidó E. Kondova I. Bontrop R. (2015). Origins of de novo genes in human and chimpanzee. *PLoS Genet.* 11:e1005721. 10.1371/journal.pgen.1005721 26720152 PMC4697840

[B76] Ruiz-Orera J. Messeguer X. Antonio Subirana J. Mar Alba M. (2014). Long non-coding RNAs as a source of new peptides. *ELife* 3:e03523. 10.7554/eLife.03523 25233276 PMC4359382

[B77] Ruiz-Orera J. Verdaguer-Grau P. Villanueva-Cañas J. L. Messeguer X. Albà M. M. (2018). Translation of neutrally evolving peptides provides a basis for de novo gene evolution. *Nat. Ecol. Evol.* 2 890–896. 10.1038/s41559-018-0506-6 29556078

[B78] Schlötterer C. (2015). Genes from scratch – the evolutionary fate of de novo genes. *Trends Genet.* 31 215–219. 10.1016/j.tig.2015.02.007 25773713 PMC4383367

[B79] Sertil O. Cohen B. D. Davies K. J. Lowry C. V. (1997). The DAN1 gene of *S. cerevisiae* is regulated in parallel with the hypoxic genes, but by a different mechanism. *Gene* 192 199–205. 10.1016/s0378-1119(97)00028-0 9224891

[B80] Šestak M. S. Domazet-Lošo T. (2015). Phylostratigraphic profiles in zebrafish uncover chordate origins of the vertebrate brain. *Mol. Biol. Evol.* 32 299–312. 10.1093/molbev/msu319 25415965 PMC4298178

[B81] Singh U. Hur M. Dorman K. Wurtele E. (2020). MetaOmGraph: a workbench for interactive exploratory data analysis of large expression datasets. *Nucleic Acids Res.* 48:e23. 10.1093/nar/gkz1209 31956905 PMC7039010

[B82] Smith J. E. Alvarez-Dominguez J. R. Kline N. Huynh N. J. Geisler S. Hu W. (2014). Translation of small open reading frames within unannotated RNA transcripts in *Saccharomyces cerevisiae*. *Cell Rep.* 7 1858–1866. 10.1016/j.celrep.2014.05.023 24931603 PMC4105149

[B83] Spellman P. T. Sherlock G. Zhang M. Q. Iyer V. R. Anders K. Eisen M. B. (1998). Comprehensive identification of cell cycle–regulated genes of the yeast *Saccharomyces cerevisiae* by microarray hybridization. *Mol. Biol. Cell* 9 3273–3297. 10.1091/mbc.9.12.3273 9843569 PMC25624

[B84] Storz G. Wolf Y. I. Ramamurthi K. S. (2014). Small proteins can no longer be ignored. *Annu. Rev. Biochem.* 83 753–777. 10.1146/annurev-biochem-070611-102400 24606146 PMC4166647

[B85] Struhl K. (2007). Transcriptional noise and the fidelity of initiation by RNA polymerase II. *Nat. Struct. Mol. Biol.* 14:103. 10.1038/nsmb0207-103 17277804

[B86] Tautz D. Domazet-Lošo T. (2011). The evolutionary origin of orphan genes. *Nat. Rev. Genet.* 12 692–702. 10.1038/nrg3053 21878963

[B87] Toll-Riera M. Bosch N. Bellora N. Castelo R. Armengol L. Estivill X. (2009). Origin of primate orphan genes: a comparative genomics approach. *Mol. Biol. Evol.* 26 603–612. 10.1093/molbev/msn281 19064677

[B88] Tukey J. W. (1977). *Exploratory Data Analysis.* Boston, MA: Addison-Wesley.

[B89] Uwimana N. Collin P. Jeronimo C. Haibe-Kains B. Robert F. (2017). Bidirectional terminators in *Saccharomyces cerevisiae* prevent cryptic transcription from invading neighboring genes. *Nucleic Acids Res.* 45 6417–6426. 10.1093/nar/gkx242 28383698 PMC5499651

[B90] Vakirlis N. Carvunis A. R. McLysaght A. (2020). Synteny-based analyses indicate that sequence divergence is not the main source of orphan genes. *Elife* 9:e53500. 10.7554/eLife.53500 32066524 PMC7028367

[B91] Vakirlis N. Hebert A. S. Opulente D. A. Achaz G. Hittinger C. T. Fischer G. (2018). A molecular portrait of de novo genes in yeasts. *Mol. Biol. Evol.* 35 631–645. 10.1093/molbev/msx315 29220506 PMC5850487

[B92] van Dongen S. M. (2000). *Graph Clustering by Flow Simulation.* Doctoral Dissertation. Utrecht: University of Utrecht, 1.

[B93] Van Oss S. B. Carvunis A. R. (2019). De novo gene birth. *PLoS Genet.* 15:e1008160. 10.1371/journal.pgen.1008160 31120894 PMC6542195

[B94] Vanderperre B. Lucier J. F. Bissonnette C. Motard J. Tremblay G. Vanderperre S. (2013). Direct detection of alternative open reading frames translation products in human significantly expands the proteome. *PLoS One* 8:e70698. 10.1371/journal.pone.0070698 23950983 PMC3741303

[B95] Weijers S. R. de Jonge J. van Zanten O. Benedetti L. Langeveld J. Menkveld H. W. (2012). KALLISTO: cost effective and integrated optimization of the urban wastewater system eindhoven. *Water Pract. Technol.* 7. 10.2166/wpt.2012.036

[B96] Werner M. S. Sieriebriennikov B. Prabh N. Loschko T. Lanz C. Sommer R. J. (2018). Young genes have distinct gene structure, epigenetic profiles, and transcriptional regulation. *Genome Res.* 28 1675–1687. 10.1101/gr.234872.118 30232198 PMC6211652

[B97] Wery M. Descrimes M. Vogt N. Dallongeville A. S. Gautheret D. Morillon A. (2016). Nonsense-mediated decay restricts LncRNA levels in yeast unless blocked by double-stranded RNA structure. *Mol. Cell* 61 379–392. 10.1016/j.molcel.2015.12.020 26805575 PMC4747904

[B98] Wilson B. A. Masel J. (2011). Putatively noncoding transcripts show extensive association with ribosomes. *Genome Biol. Evol.* 3 1245–1252. 10.1093/gbe/evr099 21948395 PMC3209793

[B99] Wu B. Knudson A. (2018). Tracing the de novo origin of protein-coding genes in yeast. *MBio* 9:e01024-18. 10.1128/mBio.01024-18 30065088 PMC6069113

[B100] Wu D. D. Irwin D. M. Zhang Y. P. (2011). De novo origin of human protein-coding genes. *PLoS Genet.* 7:e1002379. 10.1371/journal.pgen.1002379 22102831 PMC3213175

[B101] Wu H. L. Song G. Walley J. W. Hsu P. Y. (2019). The tomato translational landscape revealed by transcriptome assembly and ribosome profiling. *Plant Physiol.* 181 367–380. 10.1104/pp.19.00541 31248964 PMC6716236

[B102] Xie C. Bekpen C. Künzel S. Keshavarz M. Krebs-Wheaton R. Skrabar N. (2019). A de novo evolved gene in the house mouse regulates female pregnancy cycles. *Elife* 8:e44392. 10.7554/eLife.44392 31436535 PMC6760900

[B103] Xu Z. Wei W. Gagneur J. Perocchi F. Clauder-Münster S. Camblong J. (2009). Bidirectional promoters generate pervasive transcription in yeast. *Nature* 457 1033–1037. 10.1038/nature07728 19169243 PMC2766638

[B104] Yu G. Wang L. G. Han Y. He Q. Y. (2012). ClusterProfiler: an r package for comparing biological themes among gene clusters. *OMICS J. Integr. Biol.* 16 284–287. 10.1089/omi.2011.0118 22455463 PMC3339379

[B105] Zhang X. Zhao X. M. He K. Lu L. Cao Y. Liu J. (2012). Inferring gene regulatory networks from gene expression data by path consistency algorithm based on conditional mutual information. *Bioinformatics* 28 98–104. 10.1093/bioinformatics/btr626 22088843

[B106] Zhao S. Zhang Y. Gordon W. Quan J. Xi H. Du S. (2015). Comparison of stranded and non-stranded RNA-seq transcriptome profiling and investigation of gene overlap. *BMC Genomics* 16:675. 10.1186/s12864-015-1876-7 26334759 PMC4559181

