# Supplementary material for: Landscape of the Dark Transcriptome Revealed Through Re-mining Massive RNA-Seq Data
Source: Front Genet. 2021 Aug 16;12:722981. doi: 10.3389/fgene.2021.722981 (PMC8415361; doi:10.3389/fgene.2021.722981)
Supplement: Supplementary file 1 [file Data_Sheet_1.PDF]

Supplementary Materials

Contents

Section 1. Orphan gene identification ... 3
Section 2. Data, metadata download and RNA-Seq raw data processing. ... 4
Section 3. Comparison of normalization by *EdgeR* and *SCnorm* ... 6
Section 4. Co-expression cutoff determinations, clustering, and GO enrichment ... 9
Section 5. CDS/ORF length and GC content across phylostrata ... 12
Section 6. RNA-Seq expression for different categories of transcripts ... 14
Section 7. Ribo-Seq analysis ... 18
Section 8. Study case: Cluster317 ... 19
Section 9. Other supplementary materials available online: ... 21
Section 10. References ... 21
Section 11. Saccharomyces sequence source ... 23
Section 12. 124 species for *phylostratr* ... 26

List of Figures

Figure S1 3
Figure S2 4
Figure S3 5
Figure S4 7
Figure S5 8
Figure S6 10
Figure S7 11
Figure S8 13
Figure S9 14
Figure S10 16
Figure S11 16
Figure S12 17
Figure S13 18
Figure S14 19
Figure S15 20

List of Tables

Table S1 6
Table S2 8
Table S3 11
Table S4 15
Table S5 15

Section 1. Orphan gene identification

To stratify the phylostrata level for each gene/ORF, we used a total of 124 species, 17 of which are speices within *Saccharomycotina*. Specifically, we used 10 species with high quality genomes for the clade *Saccharomycotina*, and 7 species with high quality genomes for the clade *Saccharomyces ssp*. To account for potential incomplete gene annotation in *Saccharomyces,* we included as input data the translation products of all ORFs for each *Saccharomyces* species, as well as all the annotated proteins. Thus, if a (translated) ORF in *S. cerevisiae* had homology to a (translated) ORF, it was assigned to that clade, rather than as an orphan-ORF. This provided a stringent and more conservative determination of orphan-ORFs.

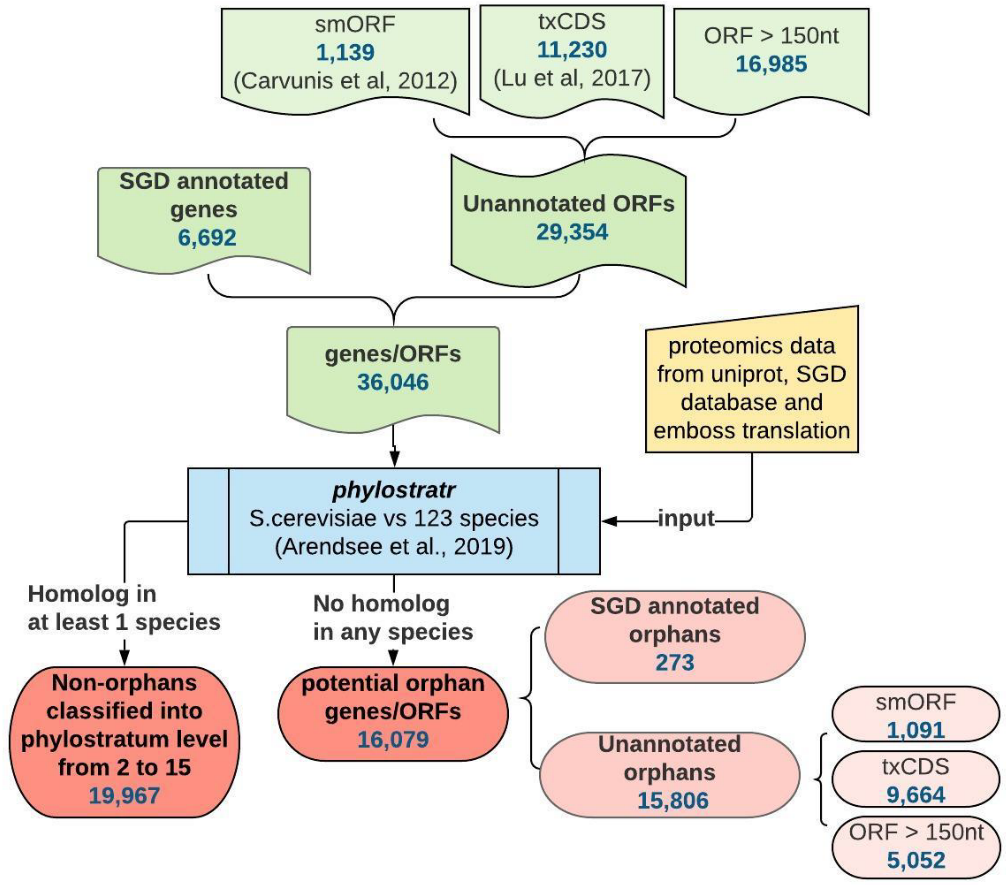

Figure S1. **Orphan-ORF detection by *phylostratr*.** The phylostrata of the 36,046 genes and ORFs in *S. cerevisiae* (determined by *phylostratr* (Arendsee et al., 2019)) by pairwise BLAST of the proteins of *S. cerevisiae* compared to other 123 species (see *species.xlsx*); E-value cutoff to infer homology, 0.001. The default NCBI clade tree was used except for species within *Saccharomyces*. For these species, we revised the clade tree manually to reflect current knowledge of yeast phylogeny (Borneman et al., 2015). Genes/ORFs were assigned to the phylostratum level associated with the deepest clade with inferred homolog. Genes/ORFs were inferred to be orphans if they had no homologs in the any of the other 123 species. Note that, for ease of comparison among the literature, throughout this study when we are specifically comparing groups of ORFS, we refer to "smORFs" (Carvunis et a., 2012) and "txCDS" (Lu et al.,2017) by the names designated in the papers that described them; the ORFs we identify in this study we designate as ORFS >150 nt. If we are not dividing into groups, we refer to them as ORFs. smORF, small ORFs with translation evidence identified by ribosomal profiling (Carvunis et al., 2012); ORFs, unannotated ORFs of ≥ 150 nt (excluding smORFs); txCDS, ORFs < 150 nt with transcription evidence identified by TIF-seq (Lu et al., 2017);

Section 2. Data, metadata download and RNA-Seq raw data processing.

The RNA-Seq data analysis workflow is shown as Figure S2. All the transcriptomic metadata and raw data were collected from public database. First, we collected SRR run IDs from (National Center for Biotechnology Information-Sequence Read Archive) (NCBI-SRA) using SRA advanced search builder. We chose all runs with *S.cerevisiae* taxon ID 4932, Illumina platform, and paired layout, and then filtered out the runs with miRNA-Seq, ncRNA-Seq, and RIP-Seq library strategy. In all, we collected 3,457 RNA-Seq runs from 177 studies. We used R packages *SRAdb* (Zhu et al., 213) and *GEOmetadb* (Zhu et al., 2008) to download RNA-Seq metadata from SRA and GEO database. *SRA toolkit* was used to download the raw RNA-Seq reads from the SRA database.

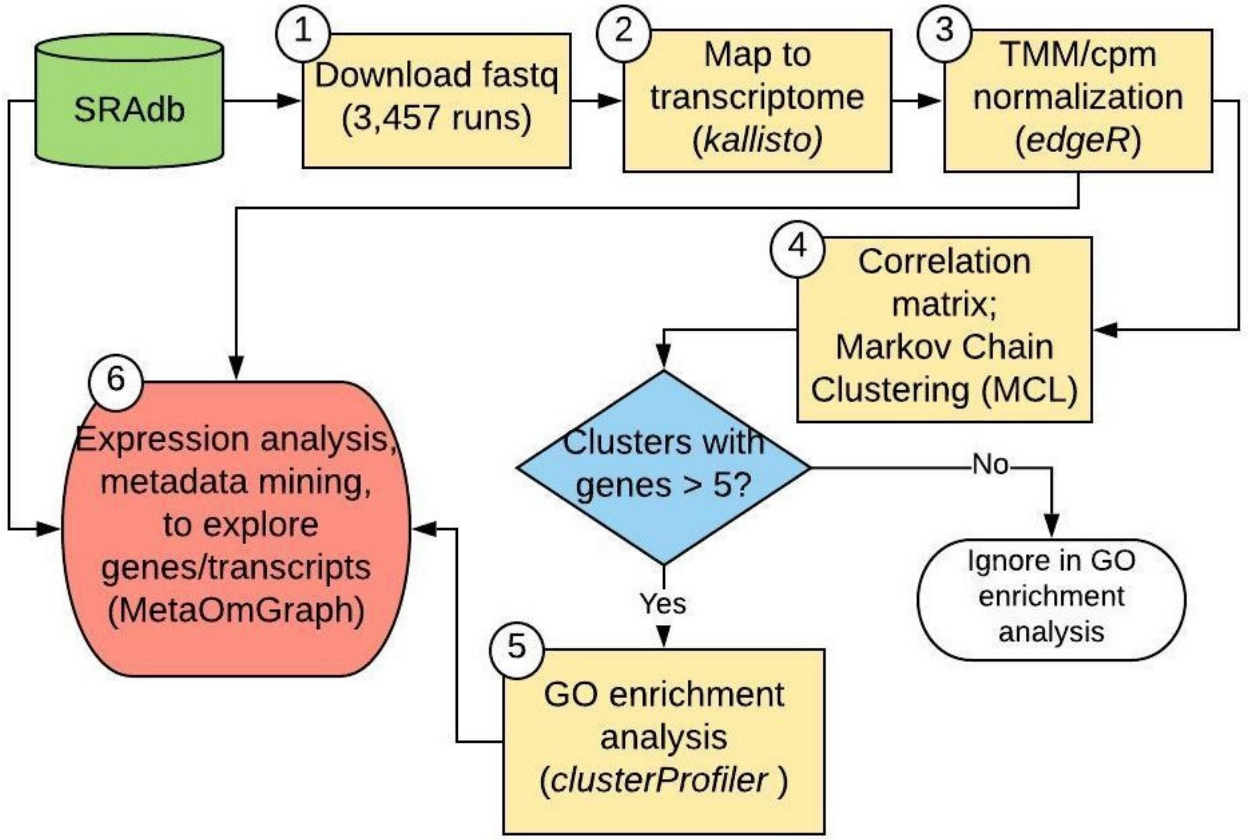

Figure S2. **RNA-Seq data analysis workflow.** 1) Raw reads and metadata for 3,457 samples (runs) of RNA-Seq were downloaded from SRA. 2) RNA-Seq reads were mapped to a transcriptome of genes and ORFs and abundance was quantified by kallisto. 3) TMM normalization (Robinson & Oshlack, 2010) by edgeR was applied to minimize technical variability of the data, with cpm as units. 4) A Pearson correlation matrix was calculated and MCL clustering analysis was performed using a home script (See code in GitHub. 5) GO enrichment analysis was conducted for clusters of five or more transcripts. 6) Full data and metadata were interactively statistically analysed and visualized in MOG (Singh et al., 2020) to explore and infer functions of selected transcripts.

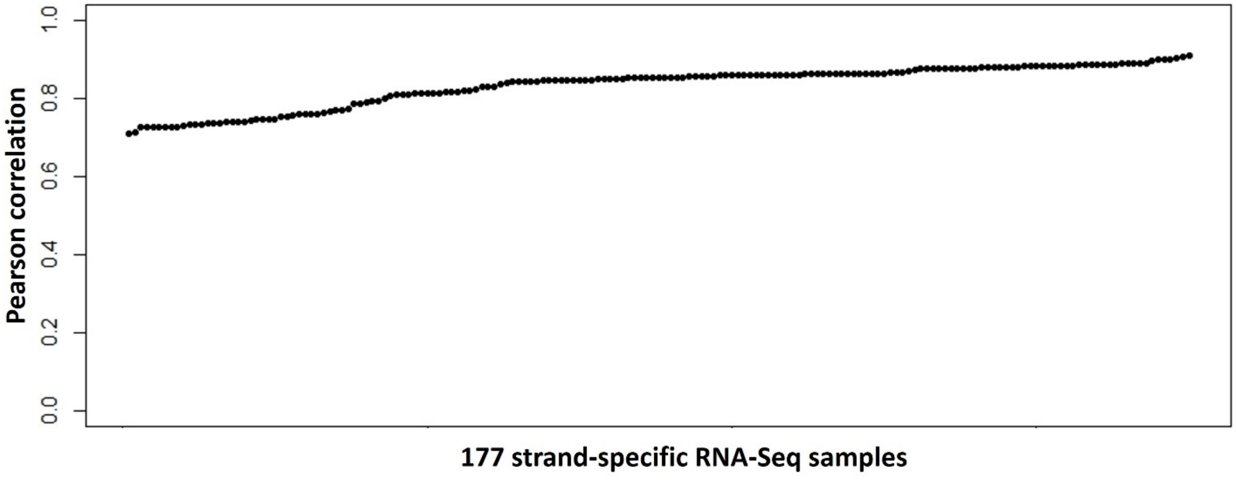

Figure S3. **Correlation of stranded and non-stranded option for strand-specific RNA-seq samples.** X-axis, 177 samples sorted by the pearson correlation. Y-axis, the pairwise pearson correlation of expression between stranded and non-stranded option for stranded RNA-Seq samples estimated by kallisto.

Section 3. Comparison of normalization by *EdgeR* and *SCnorm*

Performance of the *EdgeR* and *SCnorm* normalization methods was assessed. *EdgeR* (Robinson et al., 2010) is a commonly applied package based on TMM normalization. We used M and A values to estimate scaling factors (Robinson et al., 2010), and then used counts per million (cpm) as units. *SCnorm* (Bacher et al., 2017) is a method developed for normalization of single cell RNA-Seq data. Groups are formed based on each gene's median expression; a quantile regression was applied to estimate scaling factors, based on each gene's count-depth relationship within each group; we are not aware of *SCnorm* having been applied to "standard' RNA-Seq data.

We evaluated the *edgeR* and *SCnorm* normalization by comparing boxplot of each samples among raw counts and two normalization methods (Figure S4), creating Pearson correlation matrices from the normalized data, applying a PCC cutoff of 0.6, followed by MCL clustering and GO term enrichment analysis. After MCL analysis, *SCnorm* normalization yielded more clusters and contained more transcripts, especially more orphan transcripts in the clusters than *edgeR* (Table S1). On the downside, *SCnorm* result had more transcripts in the largest cluster, and also included more small clusters with 5-10 genes and no GO term overrepresentation assignments.

We used ARI and Jaccard indices to compare the MCL clusters obtained from raw cpm data and after *edgeR* and *SCnorm* normalization. There was only partial overlap among the clusters obtained by these two methods. Clusters from the SGD+ORF dataset were less similar than SGD dataset (Table S2). GO enrichment analysis results for the two methods indicate more significant GO terms are found after *edgeR* normalization (Table S1). *edgeR* normalization performed better than *SCnorm* based on GO enrichment analysis of MCL clusters using datasets composed of only SGD annotated genes (SGD dataset) and composed of SGD-annotated genes and all unannotated ORFs (SGD+ORF dataset) (Figure S5). Hence, for our data, *edgeR* normalization yielded the best results, and was used for subsequent analysis.

Gene list	Data	Total genes	Genes in clusters (total)	Clusters	Genes in largest cluster	Clusters with gene>5	Total orphan	Orphans in clusters (total)	Orphans in cluster with gene>5	Significant GO terms
SGD genes	Raw	6,692	6,455	49	3,564	15	384	317	297	
	edgeR	6,692	4,351	226	2,509	49	384	155	125	3,160
	scnorm	6,692	4,622	289	2,573	67	384	242	170	2,154
Combined transcripts	Raw	14,885	13,639	194	10,884	44	4,010	3,435	3,314	
	edgeR	14,885	9705	544	3,328	89	4,010	2,647	2,336	2,205
	scnorm	14,885	10,630	562	4,658	133	4,010	3,012	2,752	1,950

Table S1. **Comparison of MCL clustering results for *edgeR* and *SCnorm* normalization.** Purple font, MCL results for SGD-annotated genes. Blue font, MCL results for SGD+ORF transcripts.

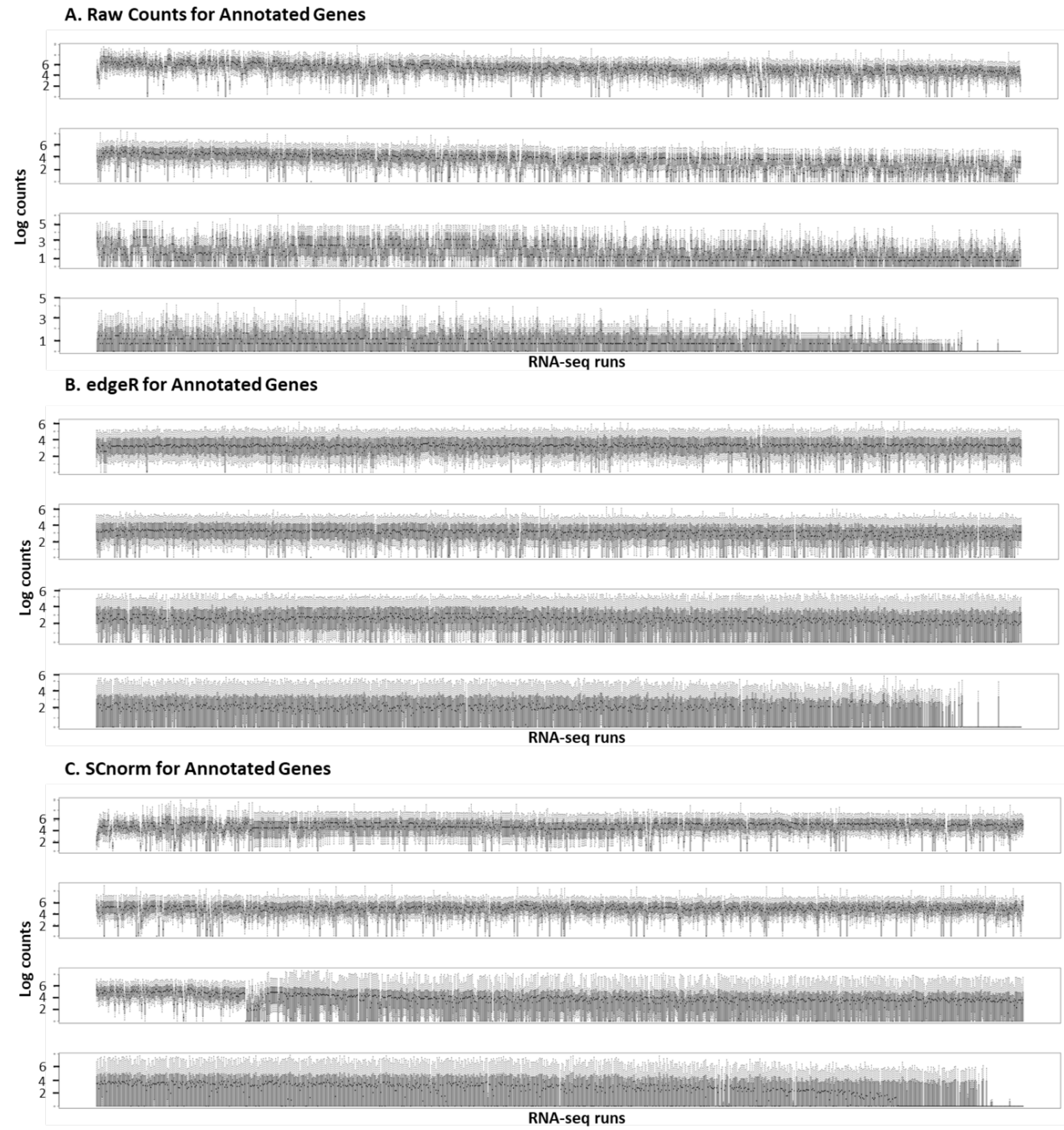

Figure S4. **Comparison of boxplots of each RNA-Seq run after *edgeR* and *SCnorm* normalization.** Values for SDG-annotated genes plotted across 3,457 samples are shown. X-axis, 3,457 RNA-Seq runs (split into 4 plots for ease of viewing). Y-axis, log transformed normalized counts. (A) Raw counts. (B) *edgeR*-normalized counts. (C) *SCnorm*-normalized counts.

Similarity index	Raw vs TMM	Raw vs SCnorm	TMM vs SCnorm
		SGD	
ARI	0.140	0.144	0.586
Jaccard index	0.249	0.243	0.529
		Combined Transcripts	
ARI	0.042	0.087	0.354
Jaccard index	0.181	0.212	0.313

Table S2. **Comparison of MCL clustering results for *edgeR* and *SCnorm* normalization using ARI and Jaccard indices.** These analyses evaluate similarity of clustering results across normalization methods. Adjusted index (ARI) (Rand et al., 1971); Jaccard index (Jaccard et al., 1901). SGD, SGD-annotated genes; Combined transcripts, SGD-annotated genes plus all ORFS (smORFs, txCDSs, + ORFs>150 nt). Raw, raw counts dataset; TMM, *EdgeR* normalization dataset; *SCnorm*, *SCnorm* normalization dataset

$$\overbrace{\widehat{ARI}}^{\text{Adjusted Index}} = \frac{\overbrace{\sum_{ij}\binom{n_{ij}}{2}}^{\text{Index}} - \overbrace{[\sum_i \binom{a_i}{2}\sum_j \binom{b_j}{2}]/\binom{n}{2}}^{\text{Expected Index}}}{\underbrace{\frac{1}{2}[\sum_i \binom{a_i}{2} + \sum_j \binom{b_j}{2}]}_{\text{Max Index}} - \underbrace{[\sum_i \binom{a_i}{2}\sum_j \binom{b_j}{2}]/\binom{n}{2}}_{\text{Expected Index}}}$$

where n_{ij}, a_i, b_j are values from the contingency table.

$$\text{Jaccard index} = \frac{|A \cap B|}{|A \cup B|} = \frac{|A \cap B|}{|A| + |B| - |A \cap B|}.$$

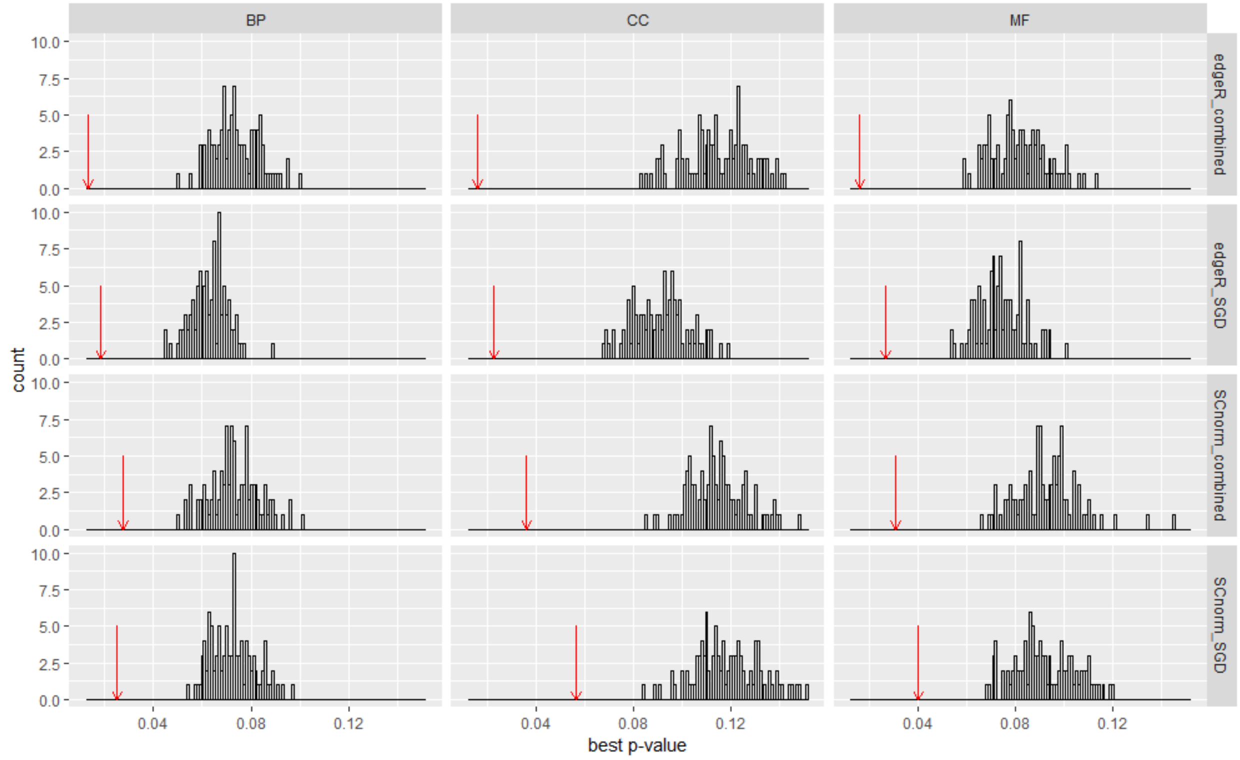

Figure S5. **Random set distribution of GO enrichment analysis for SGD genes and SGD+ORF combined transcripts for two normalization methods.** The red arrow is the best p-value for the experimental data. Random distribution, black bars, best p-value of GO terms from 100 randomly-obtained sets with the same size and number of clusters as the experimental data. Best p-value, mean of lowest adjusted p-value of GO terms across all clusters in a random set or in the experiment data (Mentzen and Wurtele, 2008).

Section 4. Co-expression cutoff determinations, clustering, and GO enrichment

We only consider the positive correlation in our network analysis. To determine a cutoff range for Pearson Correlation Coefficient (PCC), we evaluated the largest connected component and the network density for multiple PCC cutoffs (Figure S6-A). The largest connected component decreased with an increasing PCC cutoff, a linear decrease in the largest connected component occurs between 0.6 and 0.8. Network density rapidly increases when the cutoff is below 0.7, indicating that the available nodes become more densely connected with the increase in the network density below this cutoff. Thus, this method indicates a PCC cutoff of between 0.6 and 0.7. We also applied *findThreshold* function in *coexnet* (R package) (Henao et al., 2017), this method determines the optimal cutoff at 0.74 (Figure S6-B). Therefore, we tested the performance of 0.6, 0.7, and 0.8 as PCC cutoffs for the networks used in MCL clustering.

PCC co-expression matrices for the SGD and SGD+ORF datasets were transformed into a binary matrix by replacing the values of all correlations larger than the selected cutoff value by 1, and assigning the others as 0. The matrices were provided to MCL clustering software. A Spark JAVA script was used for network inference and MCL clustering (https://github.com/lijing28101/SPARK_MCL); this script is faster than R and can handle larger datasets. The power and inflation parameters for MCL analysis were set as 2.

For GO enrichment analysis of the resultant clusters, yeast genes were mapped to GO terms downloaded from the SGD database. The over-representation values for GO terms in each cluster were obtained by *enricher* in *clusterProfiler* (R package) (Yu et al., 2012); the maximal size of genes for a GO term was set as 500, and the minimal size was set as 1.

In a random test distribution of GO enrichment analysis (Mentzen and Wurtele, 2008), the experimental data has smaller p-values than any random data for networks derived using any of three cutoffs. However, cutoffs of 0.6 and 0.7 perform better, since the distance of experimental data from the random distribution is larger than for cutoff 0.8 (Figure S7). Then, we compared MCL analysis of networks based on cutoffs 0.6 and 0.7 using mean of the lowest p-value for each cluster of the experimental data (Mentzen and Wurtele, 2008), and the Z-score between the experimental data and random distribution data (Table S3). The mean of the lowest p- values for experimental data are very similar for these two cutoffs, but the Z-scores for GO molecular function (MF) and biological process (BP) of PCC 0.6 are better than those of 0.7. Moreover, PCC 0.6 cutoff resulted in more orphan genes in the clusters. Thus, we choose 0.6 as the MCL clustering cutoff for the further analysis.

A.

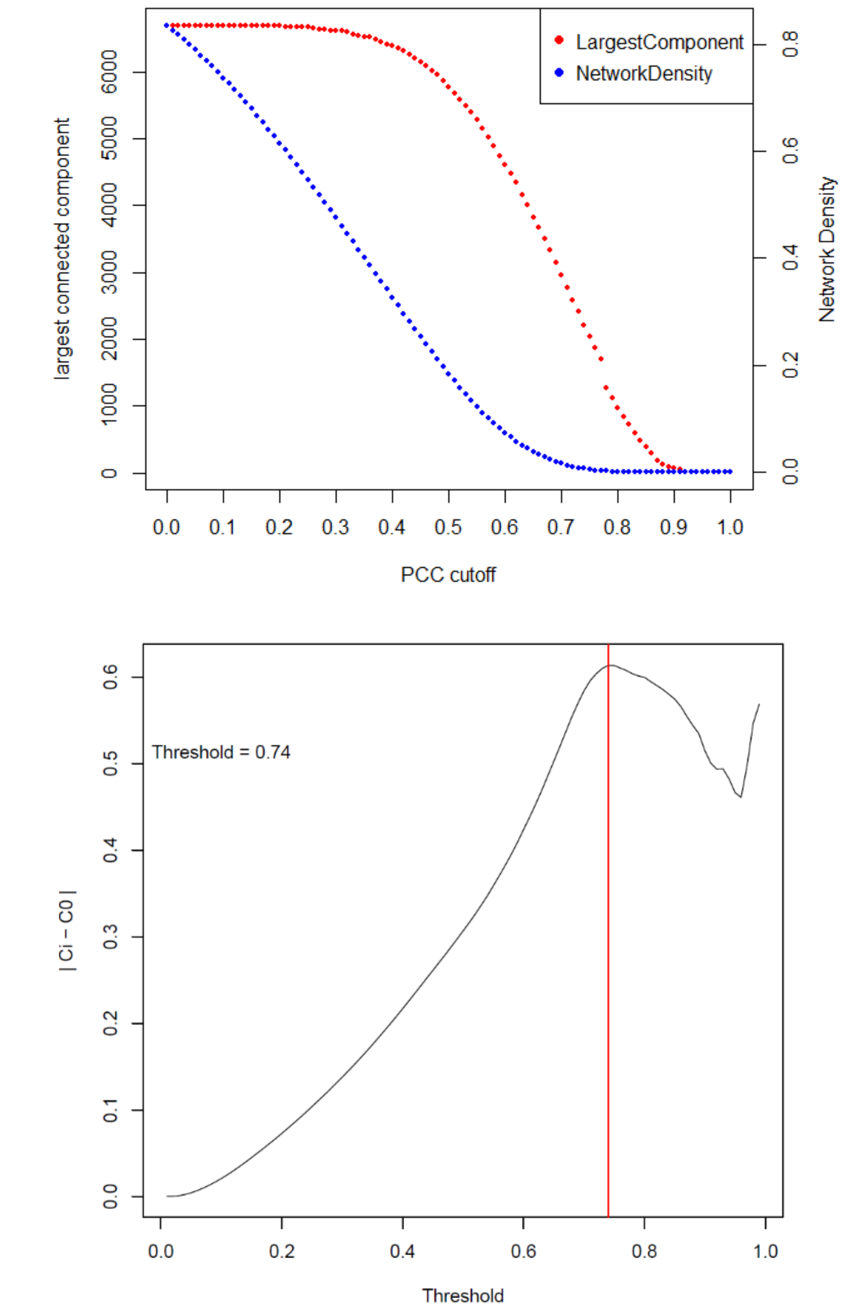

Figure S6. **Pearson correlation coefficient cutoff determination.** (A) Graph of the number of nodes in the largest connected component and network density for each Pearson correlation coefficient (PCC) cutoff. (B) The Pearson correlation coefficient cutoff determined by *coexnet* (Henao et al., 2017). X-axis, Pearson correlation coefficient (the optimized threshold is 0.74). Y-axis, the difference of clustering coefficient between the current threshold value under test and simulated random network.

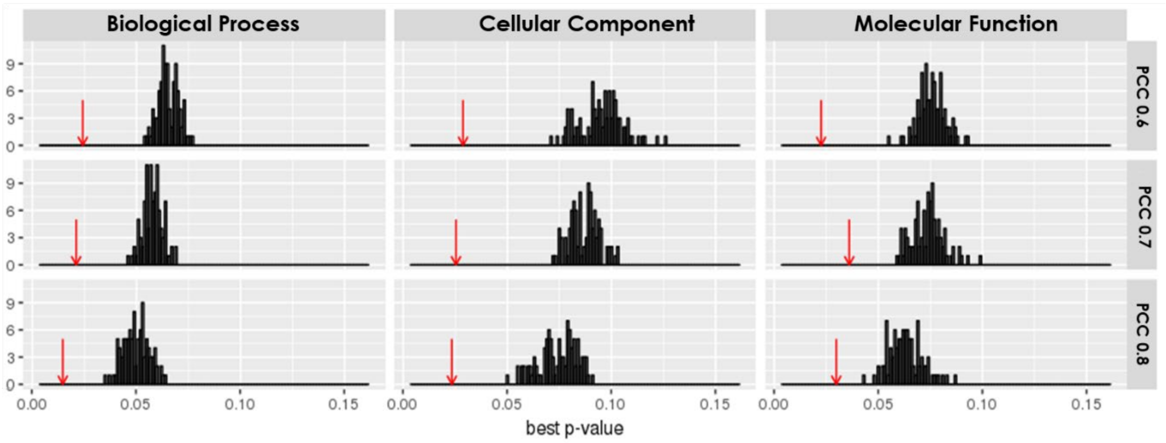

Figure S7. **Experimental versus random set distribution of GO enrichment analysis for SGD genes at three PCC cutoffs.** Red arrow, best p-value for the experimental data. Random distribution (black bars), results from 100 randomly-obtained datasets with the same size and number of clusters as the experimental data. The best p-value is the mean of best adjusted p-value of GO terms across all clusters in a random set or experiment data.

Parameter	Ontology	PCC 0.6	PCC 0.7	PCC 0.8
Mean of best p-value for experimental data	BP	0.024	0.021	0.015
	CC	0.029	0.025	0.023
	MF	0.022	0.036	0.030
Z-score* for experimental data vs random data	BP	8.31	7.85	5.70
	CC	6.43	8.76	5.56
	MF	8.16	4.96	4.24
Distance** for experimental data vs random data	BP	0.041	0.036	0.035
	CC	0.068	0.063	0.052
	MF	0.053	0.038	0.033

Table S3. **Network PCC cutoff comparison for GO enrichment analysis.** BP, biological process; CC, cellular component; MF, molecular function.

*Z-score= $\frac{|\bar{p}_{expmin} - mean(\bar{p}_{ranmin})|}{sd(\bar{p}_{ran}min)}$

**distance= $|\bar{p}_{expmin} - median(\bar{p}_{ranmin})|$

Section 5. CDS/ORF length and GC content across phylostrata

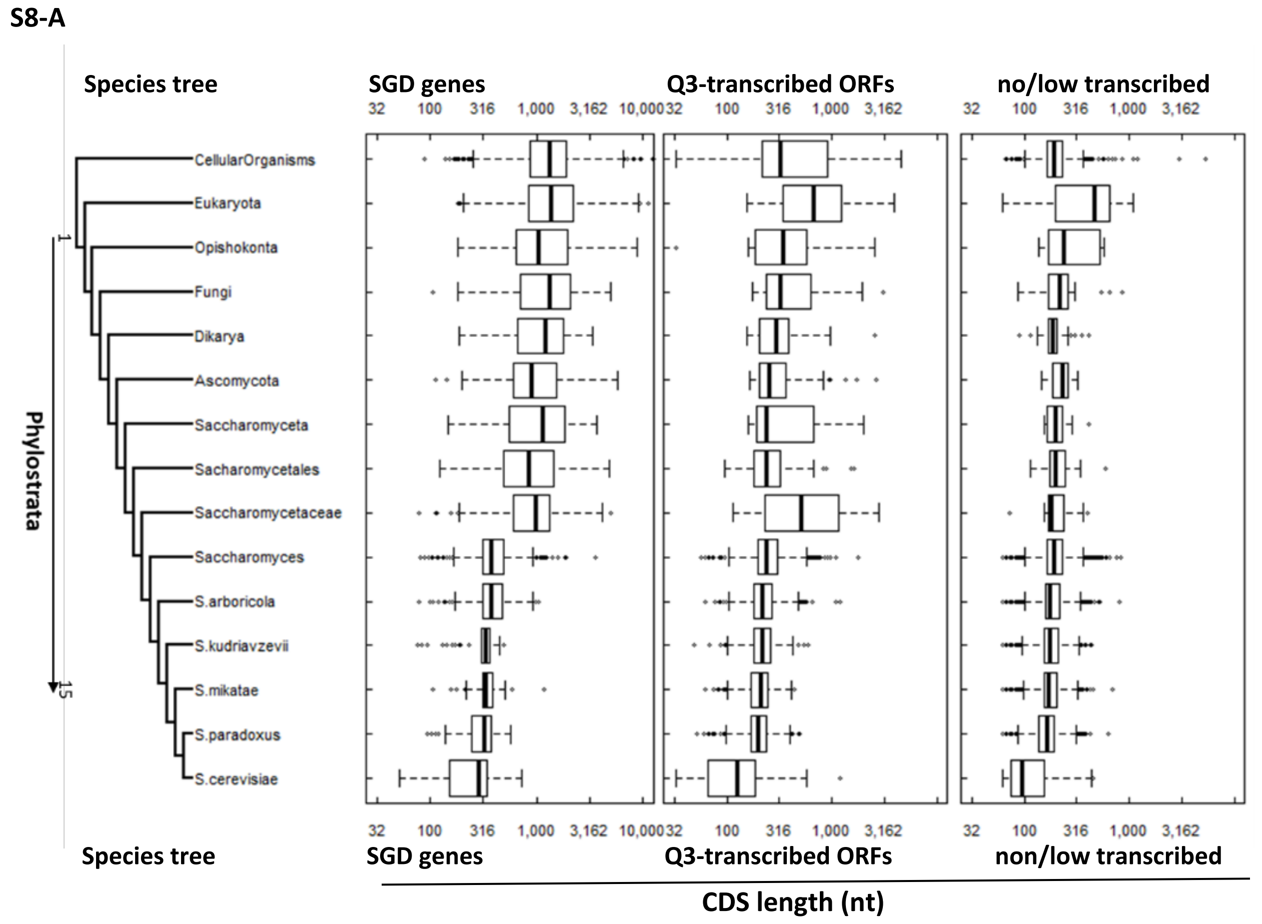

(Continues on following page)

S8-B.

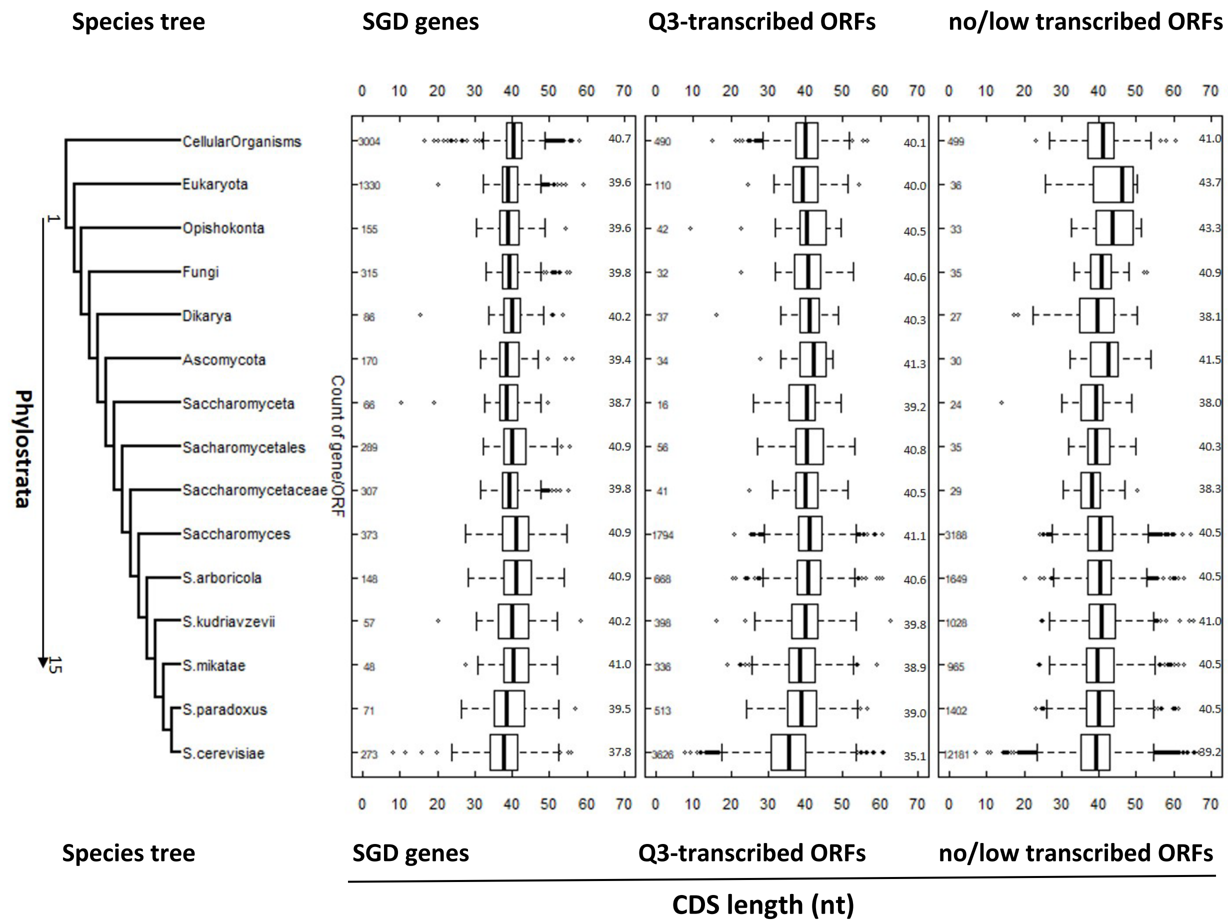

Figure S8. **CDS length and GC content of transcripts note fig 2B by phylostrata for SGD annotated genes, transcribed unannotated ORFs and non-transcribed/minimally transcribed unannotated ORFs.** From left, phylogenetic tree; first vertical panel, SGD-annotated genes (6,692); second vertical panel, Q3-transcribed ORFs, first quantile of most highly expressed unannotated ORFs (based on mean expression value/sample) (8,193); third vertical panel, no/low expressed unannotated ORFs (21,161). (A) Boxplots of CDS or ORF length. Orphan SGD genes and Q3-transcribed orphan-ORFs are significant shorter than conserved genes/ORFs. (B) Boxplots of GC content of CDS or ORF. No statistically significant for GC content between orphan and conserved genes/ORFs. The numbers of genes and ORFs in each phylostratum level are indicated in small font on left side of each vertical panel (numbers of genes and ORFs in each phylostratum level are identical for panel A and B).

Section 6. RNA-Seq expression for different categories of transcripts

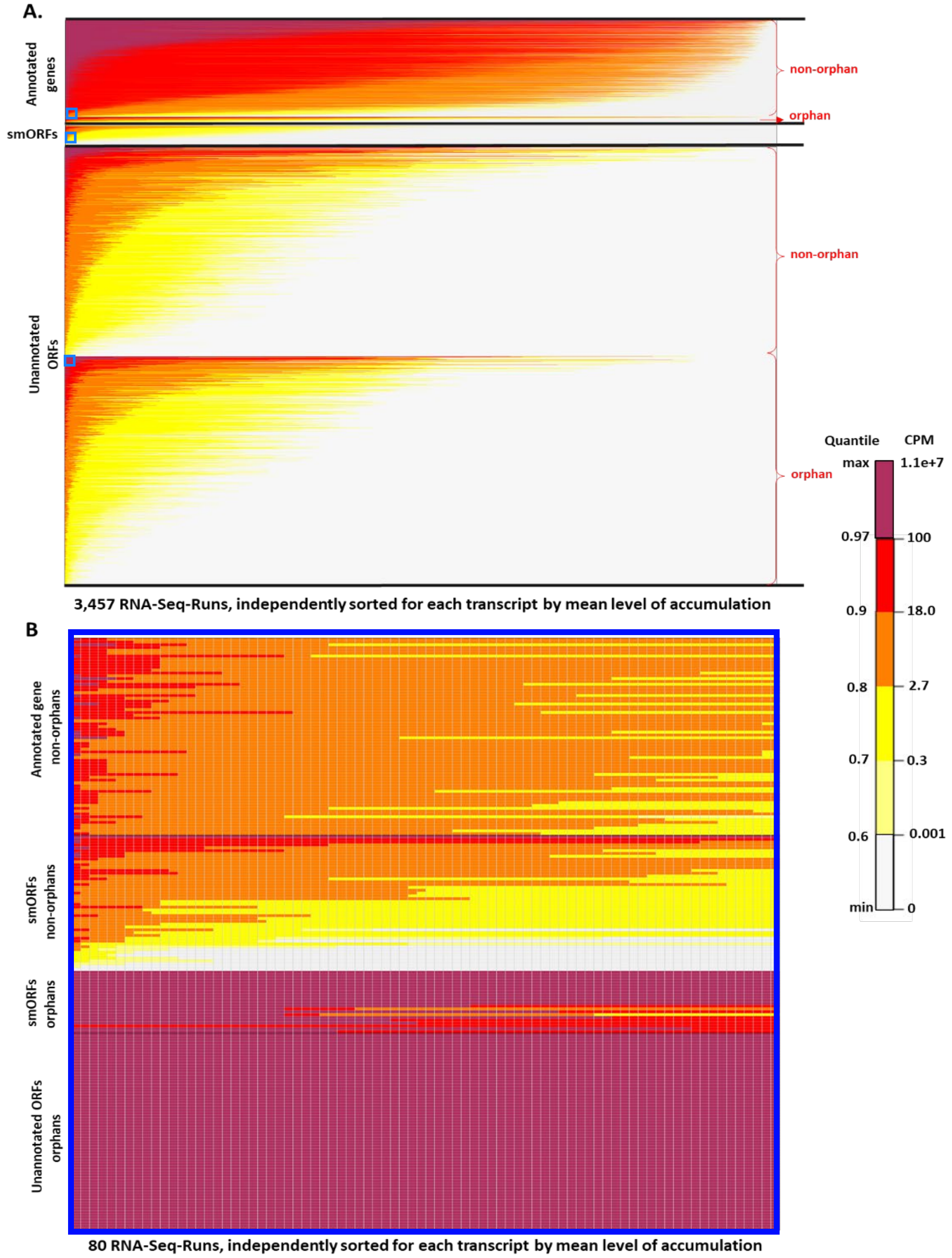

Figure S9. **RNA-Seq expression heatmap for all transcripts across 3,457 RNA-Seq runs.** Each group of transcripts is ordered independently by its mean cpm across 3,457 runs, in descending order. Each transcript is independently sorted (descending order) by cpm in sample. *EdgeR* was used for transcript normalization. (A) Heatmap of expression values for transcripts across 3,457 samples. Panels (top to bottom): 6,692 SGD-annotated genes: 6,419 non-orphan genes; 273 orphan genes; smORFs: 1,139 (Carvunis et al., 2012); all unannotated ORFs (28,215) (including 15,805 orphan-ORFs, 11,942 genus-specific ORFS, and 1,606 more highly conserved ORFS). Note: the proportion of orphans among the annotated orphan genes is very small and hard to distinguish. (B) High-resolution (blown-up) heatmap of the area *within the tiny blue boxes in panel A* (Areas marked in panel A are larger than actual area shown in panel B, for visibility). Each square corresponds to the expression value for a gene or ORF in a given sample. Panels (top to bottom): non-orphan SGD-annotated genes (#=70); non-orphan smORFs (#=48); orphan smORFs (#=22); orphan-ORFs (#=70).

To evaluate the distribution of mean expression values among the annotated genes and Q3-transcribed unannotated ORFs, we made density plots for mean expression across the 3,457 samples (Figure 4 in manuscript). We grouped SGD-annotated genes and ORFs according to their assignment to orphan versus all non-orphan ("conserved") phylostrata. The orphan-ORFs have two density peaks (Figure 4 in manuscript). When the ORFs are sub-divided further and then plotted, (Figure S10), it is clear that smORFs have lower expression other groups of ORFs. Because the smORFs are generally shorter, we evaluated whether the lower expression might be due to a bias of *kallisto* in aligning/quantifying shorter ORFs. To do this, we divided each type of ORF into four quantiles according to their length in nt, with Q1 being the shortest (Table S4). A density plot (Figure S11) shows that every length range of smORFs has a lower expression than any other group of ORFs; e.g., the mean of Q4 smORF expression is lower than any for txCDS despite that Q1 and Q2 txCDSs are shorter in length. Hence, the low expression of smORFs does not appear to be due to a technical bias in quantification of shorter reads.

A

conserved SGD-annotated genes			orphan SGD-annotated genes		all conserved-ORFs		all orphan-ORFs	
quantile(%)	mean cpm	number of genes	quantile(%)	number of genes	quantile(%)	number of ORFs	quantile(%)	number of ORFs
min	1.64E-02	6419	0.73	271	0.09	4564	7.64	3350
5	2.27E+00	6098	59.34	111	40.22	2731	58.96	1489
10	5.62E+00	5777	73.99	71	68.64	1433	81.52	671
25	1.40E+01	4814	85.71	39	83.32	763	92.06	289
50	2.71E+01	3210	90.84	25	90.10	453	95.81	153
75	5.73E+01	1605	95.97	11	94.24	264	98.01	73
90	1.31E+02	642	97.44	7	97.22	128	99.03	36
95	2.32E+02	321	99.27	2	98.55	67	99.45	21
max	3.13E+05	1	NA	0	NA	0	NA	0

B

conserved-ORFs (≥150nt)		orphan-ORFs (≥150nt)		conserved-smORF		orphan-smORF		conserved-txCDS		orphan-txCDS	
quantile(%)	number of ORFs	quantile(%)	number of ORFs	quantile(%)	number of ORFs	quantile(%)	number of ORFs	quantile(%)	number of ORFs	quantile(%)	number of ORFs
0	4386	0	1625	8.33	45	25.39	815	0	133	0	910
39.47	2655	41.29	954	81.25	10	95.51	50	48.87	68	46.26	489
68.01	1403	71.32	466	91.67	5	98.08	22	77.44	30	79.56	186
82.95	748	86.65	217	93.75	4	99.18	10	90.23	13	93.08	63
89.88	444	92.55	121	97.92	2	99.45	7	94.74	7	97.25	25
94.05	261	96.25	61	max	1	99.82	3	98.50	2	99.01	9
97.15	125	98.22	29	max	1	max	1	98.50	2	99.34	6
98.50	66	98.89	18	max	1	max	1	NA	0	99.78	2
max	1	NA	0	NA	0	NA	0	NA	0	NA	0

Table S4. **Quantile of mean expression level for each group of transcripts.** Groups of transcripts are compared relative to the quantiles of mean expression of conserved SGD-annotated genes. Mean expression level is the mean across the 3,457 RNA-Seq samples (A) Conserved SGD-annotated genes, orphan SGD-annotated genes, conserved ORFs, orphan-ORFs. (B) Subdivisions of ORFS: conserved-ORFs (≥150nt), orphan-ORFs (≥150nt), conserved-smORF, orphan-smORF, conserved-txCDS, and orphan-txCDS.

		min-25%	25%-50%	50%-75%	75%-max
smORFs	mean length (nt)	36	45	58	107
	number of ORFs	269	253	313	304
txCDSs	mean length (nt)	70	88	110	135
	number of ORFs	283	252	262	246
ORFs (≥150nt)	mean length (nt)	168	203	250	634
	number of ORFs	1,574	1,478	1505	1454
orphan-ORFs (≥150nt)	mean length (nt)	161	184	213	274
	number of ORFs	451	366	417	391
conserved-ORFs (≥150nt)	mean length (nt)	173	215	266	747
	number of ORFs	1,162	1,070	1,068	1,086

Table S5. **Quantile of mean length for each group of transcripts.** smORFs, txCDSs, and ORFs (≥150nt) were divided into quantiles according to mean length of ORF. Mean length, mean length for each quantile range; number of ORFs, number of ORFs in each quantile range.

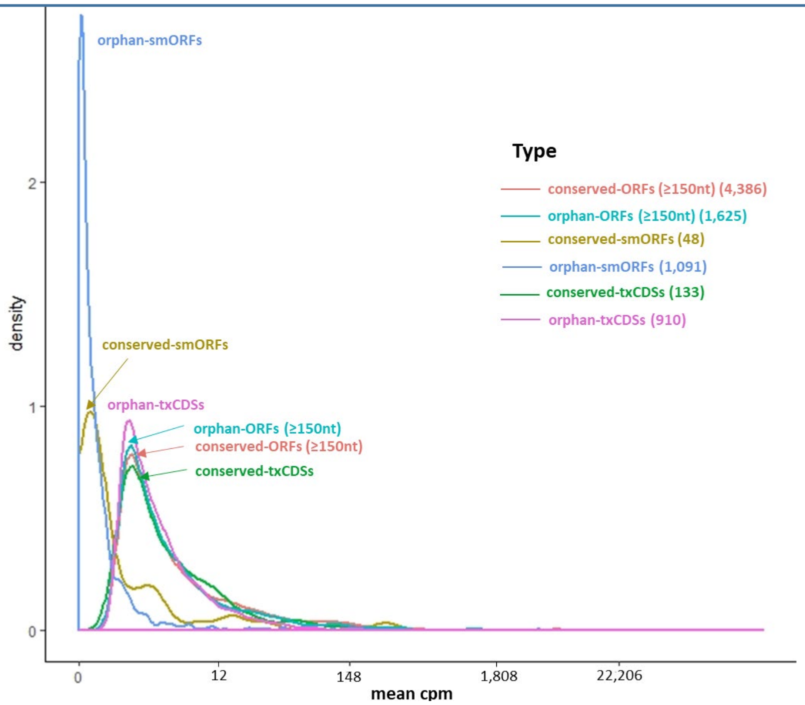

Figure S10. **Density plot for mean expression level of transcripts separated by different types of ORFs.** X-axis, *edgeR*-normalized mean expression of ORFs across 3,457 samples. The area under the curve of the density function represents the probability of a range of mean cpm.

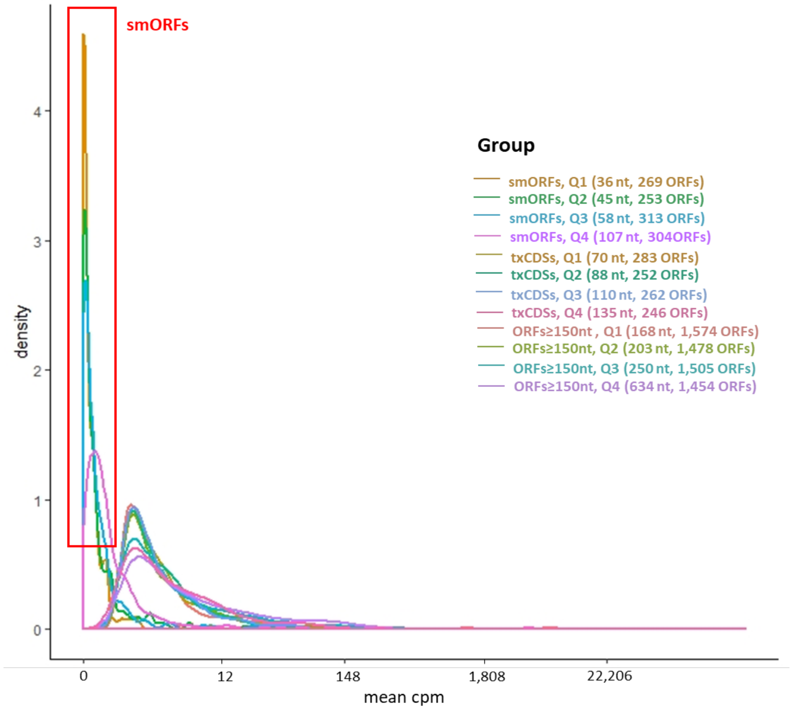

Figure S11. **Density plot showing mean expression level for each length range of different groups of ORFs.** X-axis, *edgeR*-normalized mean expression of transcripts in cpm across 3,457 samples. The area under the curve of density function represents the probability of a range of mean cpm. ORFs are classified by length: Q1 to Q4 refers to the range of length in nt, from short (Q1) to long (Q4). ORF mean length (nt) and number of ORFs in that category are given in parentheses.

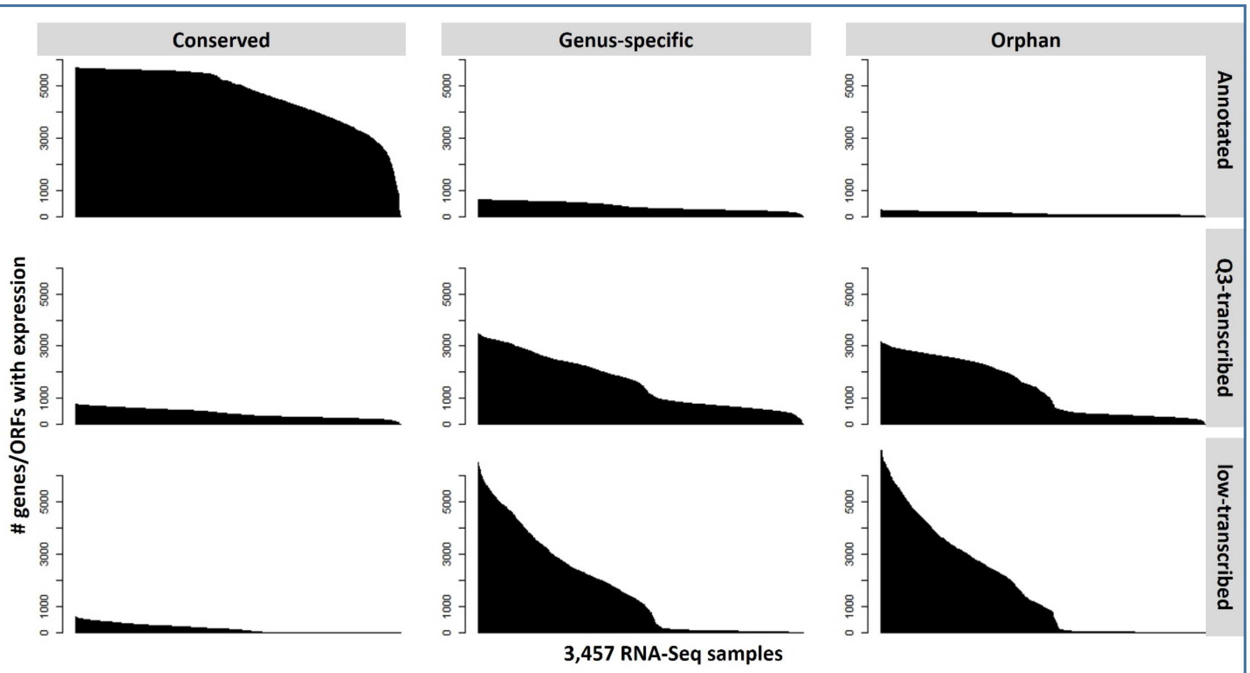

Figure S12. **Number of genes/ORFs with expression evidence in each RNA-Seq sample for different combination of group.** The black bars show distribution of the counts of genes/ORFs. X-axis, 3,457 RNA-Seq samples, sorted by counts. Y-axis, number of genes/ORFs with expression in each sample.

Section 7. Ribo-Seq analysis

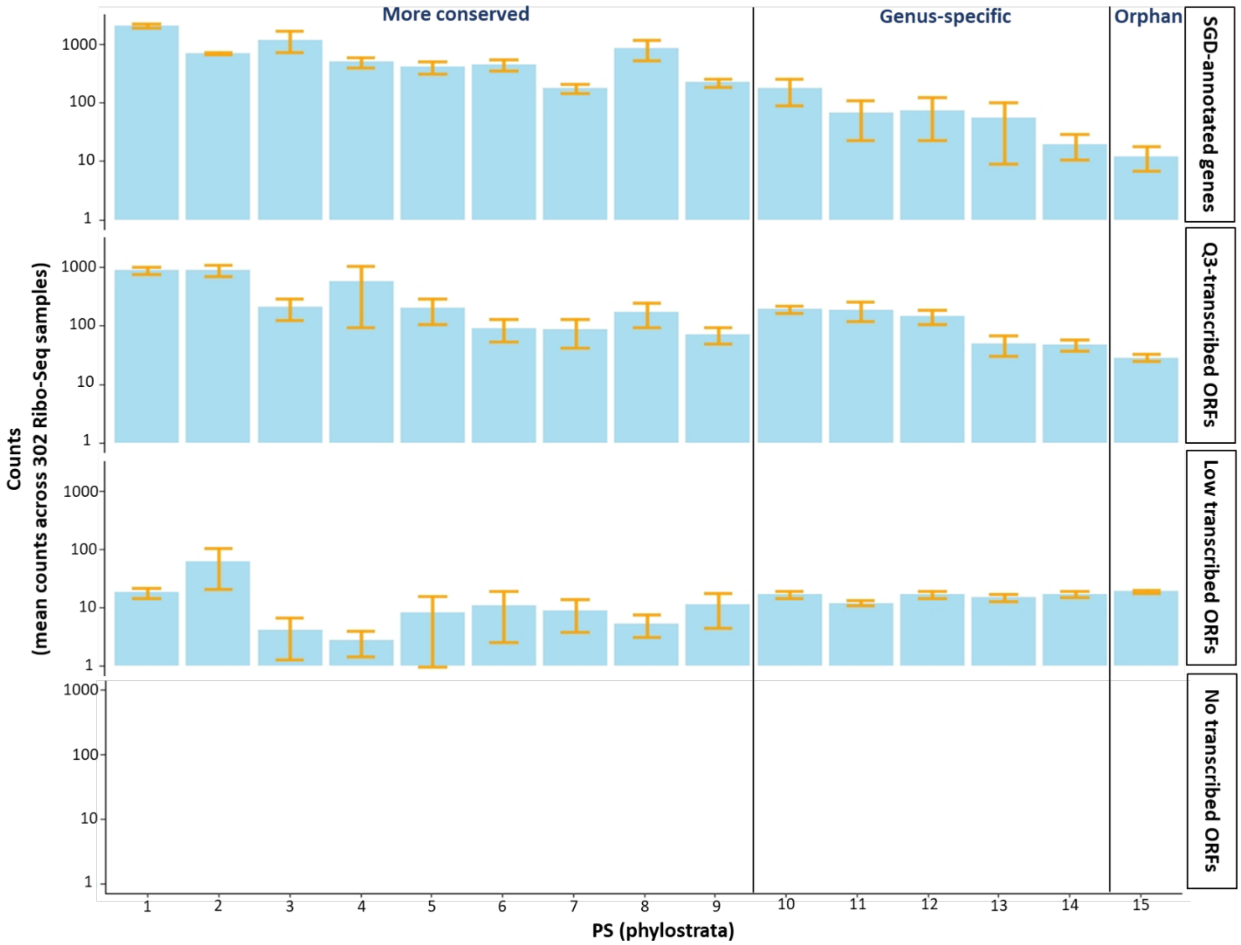

Figure S13. **Mean raw counts across 302 Ribo-Seq samples for different groups of genes/ORFs.** Translation evidence for the Q3 ORFs is similar in counts to that of the annotated genes. X-axis, PS (phylostrata) from 1 to 15 means from ancient to orphan. Orphan, PS=15; genus-specific, PS=10-14; more conserved, PS=1-9. Y-axis, mean raw counts across 302 Ribo-Seq samples for each group, the y-axis for no transcribed ORFs is different than other three groups. Yellow segment, standard error bar. An ORF is considered to have translation evidence if the mean value is greater than 0.3. Note: only 49 ORFs are non-transcribed, all in PS=15. Their mean count is less than 1, and the bar is too small to shown in the figure.

Section 8. Study case: Cluster317

Cluster317 contains 25 highly conserved (phylostratum level 1 or 2) SGD-annotated genes and 5 *Saccharomyces*-specific ORFs. Twenty-three of the 25 conserved genes encode ribosomal proteins (P1 Alpha, large, or small subunits). Another gene encodes a zuotin, a ribosome-associated chaperone that functions in ribosome biogenesis. The remaining gene encodes guanylate kinase; a gene with nine edges in common with the other genes in this cluster of the co-expression network. In red alga, guanylate kinase and chloroplastic ribosomal proteins are co-regulated by the regulatory kinase, TOR, although the reason for this is not understood (Imamura et al., 2018). Each of the five ORFs share many highly correlated edges with other transcripts (Figure S14, left). GO enrichment analysis identified nine highly over-represented GO terms (Figure S14, right) each relating to ribosomes or translation. The expression pattern of the genes in Cluster317 is similar across the 3,457 RNA-Seq runs (Figure S15). The top panel of Figure S15 shows the expression level of Cluster317 in study ERP008497, which compares wild type and temperature-sensitive ubc9-1 mutant yeast in a DMSO control group vs. rapamycin treatment (Chymkowitch et al., 2015). In yeast, TOR, regulates ribosome biogenesis , along with cell proliferation, mRNA translation, responses to nutrients, autophagy, and mating (Cardenas et al., 1999). Thus, rapamycin represses the expression of yeast ribosomal protein genes. All the transcripts in this cluster have decreased expression following rapamycin treatment, irrespective of the yeast genotype. Combining this information, the five ORFs in Cluster317 might encode proteins associated with the ribosome, or be involved in the translation process.

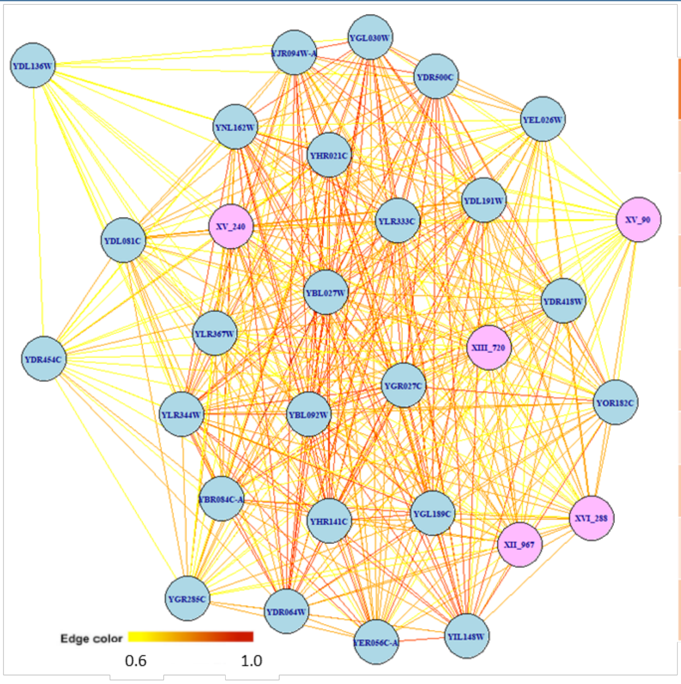

Ontology	GO name	Adjust p-value
BP	cytoplasmic translation	3.49E-32
MF	structural constituent of ribosome	4.73E-29
CC	ribosome	2.17E-28
CC	intracellular ribonucleoprotein complex	2.17E-28
CC	cytosolic large ribosomal subunit	1.32E-23
BP	translation	1.53E-22
CC	intracellular	3.03E-20
CC	cytosolic small ribosomal subunit	1.33E-08
CC	preribosome, large subunit precursor	7.12E-08

Figure S14. **Co-expression network and significant GO terms for Cluster317.** Cluster317 is composed of ribosomal related genes and five ORFs. Left, the network of Cluster317. Edge color, yellow to red, maps to Pearson correlations of 0.6 to 1. Light blue nodes, SGD-annotated genes; pink nodes, unannotated ORFs. Right, significantly enriched GO terms in Cluster317. Twenty-four of the 25 SGD-annotated genes in this cluster are assigned to GO terms in this table.

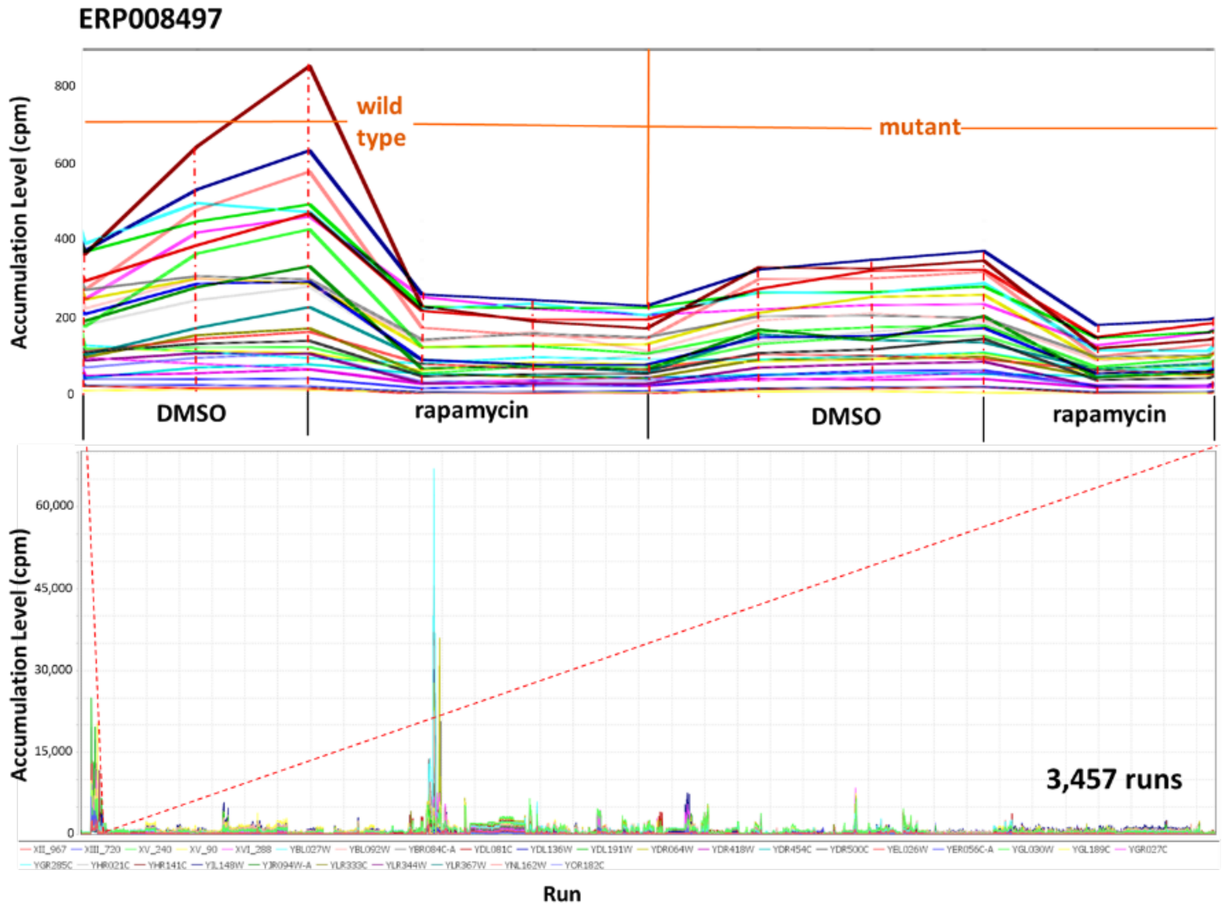

Figure S15. **The 41 genes and ORFs in Cluster317 are repressed in response to rapamycin.** Bottom panel, 3,457-samples. Expression pattern are similar for all transcripts. Top panel, high resolution of Study ERP008497: "Sumoylation of Rap1 mediates recruitment of the basal transcription machinery to promote transcription of Ribosomal Protein genes". Dimethyl sulfoxide (DMSO), control treatment. Rapamycin could repress expression of ribosomal protein.

Section 9. Other supplementary materials available online:

All of the supplementary data (include mog files, cluster information, UTR results, phylostratr heatmap, Ribo-Seq metadata and results): https://datahub.io/lijing28101/yeast_supplementary

MOG file of *Sacchromyces cerevisea* RNA-Seq expression (*S.cerevisiae_RNA-seq_3457_27.mog*): http://metnetweb.gdcb.iastate.edu/MetNet_MetaOmGraph.htm

MetaOmGraph software: https://github.com/urmi-21/MetaOmGraph

Data processing code: https://github.com/lijing28101/yeast_supplementary

Section 10. References

Bacher, Rhonda, et al. "SCnorm: robust normalization of single-cell RNA-seq data." *Nature methods* 14.6 (2017): 584.

Borneman, Anthony R., and Isak S. Pretorius. "Genomic insights into the Saccharomyces sensu stricto complex." *Genetics* 199.2 (2015): 281-291.

Cardenas, Maria E., et al. "The TOR signaling cascade regulates gene expression in response to nutrients." *Genes & development* 13.24 (1999): 3271-3279.

Carvunis, Anne-Ruxandra, et al. "Proto-genes and de novo gene birth." *Nature* 487.7407 (2012): 370.

Chymkowitch, Pierre, et al. "Sumoylation of Rap1 mediates the recruitment of TFIID to promote transcription of ribosomal protein genes." *Genome research* 25.6 (2015): 897-906.

Imamura, Sousuke, et al. "The checkpoint kinase TOR (target of rapamycin) regulates expression of a nuclear‐encoded chloroplast RelA‐SpoT homolog (RSH) and modulates chloroplast ribosomal RNA synthesis in a unicellular red alga." The Plant Journal 94.2 (2018): 327-339.

Jaccard, P. (1901) Distribution de la flore alpine dans le bassin des Dranses et dans quelques régions voisines. Bulletin de la Société Vaudoise des Sciences Naturelles 37, 241-272.

Lu, Tzu-Chiao, Jun-Yi Leu, and Wen-Chang Lin. "A comprehensive analysis of transcript-supported de novo genes in Saccharomyces sensu stricto yeasts." *Molecular biology and evolution* 34.11 (2017): 2823-2838.

Mentzen, Wieslawa I., and Eve Syrkin Wurtele. "Regulon organization of Arabidopsis." *BMC plant biology* 8.1 (2008): 99.

Rand, William M. "Objective criteria for the evaluation of clustering methods." *Journal of the American Statistical association* 66.336 (1971): 846-850.

Robinson, Mark D., Davis J. McCarthy, and Gordon K. Smyth. "edgeR: a Bioconductor package for differential expression analysis of digital gene expression data." *Bioinformatics* 26.1 (2010): 139-140.

Vera JM, Dowell RD. Survey of cryptic unstable transcripts in yeast. *BMC Genomics*; 17. Epub ahead of print 26 April 2016. DOI: 10.1186/s12864-016-2622-5.

Weijers, S. R., et al. "KALLISTO: cost effective and integrated optimization of the urban wastewater system Eindhoven." *Water Practice and Technology* 7.2 (2012).

Xu Z, Wei W, Gagneur J, et al. Bidirectional promoters generate pervasive transcription in yeast. *Nature* 2009; 457: 1033–1037.

Yu, Guangchuang, et al. "clusterProfiler: an R package for comparing biological themes among gene clusters." *Omics: a journal of integrative biology* 16.5 (2012): 284-287.

Zebulun Arendsee, Jing Li, Urminder Singh, Arun Seetharam, Karin Dorman, Eve Syrkin Wurtele, phylostratr: a framework for phylostratigraphy, *Bioinformatics*

Zhu, Yuelin, et al. "GEOmetadb: powerful alternative search engine for the Gene Expression Omnibus." *Bioinformatics* 24.23 (2008): 2798-2800.

Zhu, Yuelin, et al. "SRAdb: query and use public next-generation sequencing data from within R." BMC *Bioinformatics* 14.1 (2013): 19.

Section 11. Saccharomyces sequence source

**file**	**download date**	**release date**	**version**
Saccharomyces cerevisiae R64 gene model GFF3	10/17/2017	7/22/2017	R64-1-1
Saccharomyces cerevisiae R64 genomic sequence	10/17/2017	7/22/2017	R64-1-1
Saccharomyces cerevisiae amino acid	10/19/2017	7/22/2017	R64-1-1
smORF annotation	10/19/2017	7/19/2012	R56
Saccharomyces cerevisiae R56 genomic sequence	10/19/2017	4/6/2007	R56
Saccharomyces arboricola genomic sequence	10/19/2017	1/15/2015	SacArb1.0
Saccharomyces arboricola protein sequence	10/19/2017	1/15/2015	SacArb1.0
Saccharomyces bayanus genomic sequence	10/19/2017	6/15/2016	ASM16703v1
Saccharomyces bayanus protein sequence			
Saccharomyces eubayanus genomic sequence	10/19/2017	7/30/2017	SEUB3.0
Saccharomyces eubayanus protein sequence	10/19/2017	7/30/2017	SEUB3.0
Saccharomyces kudriavzevii genomic sequence	10/19/2017	8/4/2014	IFO1802_v1.0
Saccharomyces kudriavzevii protein sequence	10/19/2017	6/15/2016	IFO1802_v1.0
Saccharomyces mikatae genomic sequence	10/19/2017	8/1/2014	ASM16697v1
Saccharomyces mikatae protein sequence			
Saccharomyces paradoxus genomic sequence	10/19/2017	6/15/2016	ASM16695v1
Saccharomyces paradoxus protein sequence			
Saccharomyces uvarum genomic sequence	10/19/2017	6/15/2016	ASM16699v1
Saccharomyces uvarum protein sequence			

**file**	**source**
Saccharomyces cerevisiae R64 gene model GFF3	ftp://ftp.ensembl.org/pub/release-90/gff3/saccharomyces_cerevisiae/Saccharomyces_cerevisiae.R64-1-1.90.gff3.gz
Saccharomyces cerevisiae R64 genomic sequence	ftp://ftp.ensembl.org/pub/release-90/fasta/saccharomyces_cerevisiae/dna/Saccharomyces_cerevisiae.R64-1-1.dna_sm.toplevel.fa.gz
Saccharomyces cerevisiae amino acid	ftp://ftp.ensembl.org/pub/release-90/fasta/saccharomyces_cerevisiae/pep/Saccharomyces_cerevisiae.R64-1-1.pep.all.fa.gz
smORF annotation	Carvunis at al., 2012

Saccharomyces cerevisiae R56 genomic sequence	https://downloads.yeastgenome.org/sequence/S288C_reference/genome_releases/S288C_reference_genome_R56-1-1_20070406.tgz
Saccharomyces arboricola genomic sequence	ftp://ftp.ncbi.nlm.nih.gov/genomes/all/GCF/000/292/725/GCF_000292725.1_SacArb1.0/GCF_000292725.1_SacArb1.0_genomic.fna.gz
Saccharomyces arboricola protein sequence	ftp://ftp.ncbi.nlm.nih.gov/genomes/all/GCF/000/292/725/GCF_000292725.1_SacArb1.0/GCF_000292725.1_SacArb1.0_protein.faa.gz
Saccharomyces bayanus genomic sequence	ftp://ftp.ncbi.nih.gov/genomes/genbank/fungi/Saccharomyces_bayanus/latest_assembly_versions/GCA_000167035.1_ASM16703v1/GCA_000167035.1_ASM16703v1_genomic.fna.gz
Saccharomyces bayanus protein sequence	Annotated and created by Braker
Saccharomyces eubayanus genomic sequence	ftp://ftp.ncbi.nih.gov/genomes/genbank/fungi/Saccharomyces_eubayanus/latest_assembly_versions/GCA_001298625.1_SEUB3.0/GCA_001298625.1_SEUB3.0_genomic.fna.gz
Saccharomyces eubayanus protein sequence	ftp://ftp.ncbi.nih.gov/genomes/genbank/fungi/Saccharomyces_eubayanus/latest_assembly_versions/GCA_001298625.1_SEUB3.0/GCA_001298625.1_SEUB3.0_protein.faa.gz
Saccharomyces kudriavzevii genomic sequence	ftp://ftp.ncbi.nih.gov/genomes/genbank/fungi/Saccharomyces_kudriavzevii/latest_assembly_versions/GCA_000167075.2_Saccharomyces_kudriavzevii_strain_IFO1802_v1.0/GCA_000167075.2_Saccharomyces_kudriavzevii_strain_IFO1802_v1.0_genomic.fna.gz
Saccharomyces kudriavzevii protein sequence	ftp://ftp.ncbi.nih.gov/genomes/genbank/fungi/Saccharomyces_kudriavzevii/latest_assembly_versions/GCA_000167075.2_Saccharomyces_kudriavzevii_strain_IFO1802_v1.0/GCA_000167075.2_Saccharomyces_kudriavzevii_strain_IFO1802_v1.0_protein.faa.gz
Saccharomyces mikatae genomic sequence	ftp://ftp.ncbi.nih.gov/genomes/genbank/fungi/Saccharomyces_mikatae/latest_assembly_versions/GCA_000166975.1_ASM16697v1/GCA_000166975.1_ASM16697v1_genomic.fna.gz
Saccharomyces mikatae protein sequence	Annotated and created by Braker
Saccharomyces paradoxus genomic sequence	ftp://ftp.ncbi.nih.gov/genomes/genbank/fungi/Saccharomyces_paradoxus/latest_assembly_versions/GCA_000166955.1_ASM16695v1/GCA_000166955.1_ASM16695v1_genomic.fna.gz
Saccharomyces paradoxus protein sequence	Annotated and created by Braker

Saccharomyces uvarum genomic sequence	ftp://ftp.ncbi.nih.gov/genomes/genbank/fungi/Saccharomyces_uvarum/latest_assembly_versions/GCA_000166995.1_ASM16699v1/GCA_000166995.1_ASM16699v1_genomic.fna.gz
Saccharomyces uvarum protein sequence	Annotated and created by Braker

Section 12. 124 species for *phylostratr*

Species	PS	Phylostratum name
Aliifodinibius roseus	1	cellular organisms
Caldithrix sp. RBG_13_44_9	1	cellular organisms
Aminobacterium colombiense DSM 12261	1	cellular organisms
Treponema brennaborense DSM 12168	1	cellular organisms
Thermodesulfatator indicus DSM 15286	1	cellular organisms
Desulfurispirillum indicum S5	1	cellular organisms
Denitrovibrio acetiphilus DSM 12809	1	cellular organisms
Mesotoga prima	1	cellular organisms
Thermocrinis albus DSM 14484	1	cellular organisms
Elusimicrobium minutum Pei191	1	cellular organisms
Dictyoglomus turgidum DSM 6724	1	cellular organisms
Caldisericum exile AZM16c01	1	cellular organisms
Chloracidobacterium thermophilum B	1	cellular organisms
Candidatus Solibacter usitatus Ellin6076	1	cellular organisms
Nitrospira japonica	1	cellular organisms
Fusobacterium mortiferum ATCC 9817	1	cellular organisms
Hydrogenophilaceae bacterium CG1_02_62_390	1	cellular organisms
Acidithiobacillales bacterium SM1_46	1	cellular organisms
Bdellovibrionales bacterium GWA2_49_15	1	cellular organisms
Mariprofundus ferrooxydans PV−1	1	cellular organisms
Amantichitinum ursilacus	1	cellular organisms
Alphaproteobacteria bacterium RIFCSPHIGHO2_02_FULL_46_13	1	cellular organisms
Rhodanobacter sp. B04	1	cellular organisms
Acholeplasma laidlawii PG−8A	1	cellular organisms
Solirubrobacterales bacterium 67−14	1	cellular organisms
Collinsella sp. CAG:289	1	cellular organisms
Rubrobacter xylanophilus DSM 9941	1	cellular organisms
Ferrimicrobium acidiphilum DSM 19497	1	cellular organisms
Streptomyces cattleya NRRL 8057 = DSM 46488	1	cellular organisms
Ardenticatena maritima	1	cellular organisms
Caldilinea aerophila DSM 14535 = NBRC 104270	1	cellular organisms
Ktedonobacter sp. 13_2_20CM_2_56_8	1	cellular organisms
Dehalococcoidia bacterium SM23_28_2	1	cellular organisms
Longilinea arvoryzae	1	cellular organisms
Sphaerobacter thermophilus DSM 20745	1	cellular organisms
Roseiflexus sp. RS−1	1	cellular organisms
Fimbriimonas ginsengisoli Gsoil 348	1	cellular organisms
Chthonomonas calidirosea T49	1	cellular organisms
Deinococcus peraridilitoris DSM 19664	1	cellular organisms
Peptoniphilus asaccharolyticus DSM 20463	1	cellular organisms

Species	PS	Phylostratum name
Limnochorda pilosa	1	cellular organisms
Dialister micraerophilus DSM 19965	1	cellular organisms
Eubacterium sp. CAG:252	1	cellular organisms
Streptococcus dysgalactiae subsp. equisimilis RE378	1	cellular organisms
Gemmatimonadaceae bacterium 4484_173	1	cellular organisms
Chitinispirillum alkaliphilum	1	cellular organisms
Chitinivibrio alkaliphilus ACht1	1	cellular organisms
Fibrobacter sp. UWH6	1	cellular organisms
Lentisphaera araneosa HTCC2155	1	cellular organisms
Criblamydia sequanensis CRIB−18	1	cellular organisms
Phycisphaera mikurensis NBRC 102666	1	cellular organisms
Paludisphaera borealis	1	cellular organisms
Kiritimatiella glycovorans	1	cellular organisms
Methylacidiphilum infernorum V4	1	cellular organisms
Coraliomargarita sp. CAG:312	1	cellular organisms
Pedosphaera parvula Ellin514	1	cellular organisms
Chthoniobacter flavus Ellin428	1	cellular organisms
Campylobacter concisus	1	cellular organisms
Archangium gephyra	1	cellular organisms
Ignavibacteria bacterium RBG_16_36_9	1	cellular organisms
Chlorobium ferrooxidans DSM 13031	1	cellular organisms
Phaeodactylibacter xiamenensis	1	cellular organisms
Chitinophaga eiseniae	1	cellular organisms
Cytophagaceae bacterium SCN 52−12	1	cellular organisms
Porphyromonas gingivalis W83	1	cellular organisms
Sphingobacterium spiritivorum ATCC 33861	1	cellular organisms
Flavobacterium sp. A45	1	cellular organisms
Theionarchaea archaeon DG−70	1	cellular organisms
Hadesarchaea archaeon DG−33−1	1	cellular organisms
Methanocella arvoryzae MRE50	1	cellular organisms
Methanopyrus kandleri AV19	1	cellular organisms
Archaeoglobus sulfaticallidus PM70−1	1	cellular organisms
Pyrococcus furiosus DSM 3638	1	cellular organisms
methanogenic archaeon ISO4−H5	1	cellular organisms
Natrinema pellirubrum DSM 15624	1	cellular organisms
Methanococcus aeolicus Nankai−3	1	cellular organisms
Methanothermobacter thermautotrophicus str. Delta H	1	cellular organisms
Candidatus Methanohalarchaeum thermophilum	1	cellular organisms
Candidatus Nitrososphaera gargensis Ga9.2	1	cellular organisms
Thermofilum adornatus	1	cellular organisms
Aphanomyces astaci	2	Eukaryota
Oryza sativa Indica Group	2	Eukaryota

Species	PS	Phylostratum name
Leishmania donovani BPK282A1	2	Eukaryota
Acanthisitta chloris	3	Opisthokonta
Gorilla gorilla gorilla	3	Opisthokonta
Poecilia formosa	3	Opisthokonta
Anopheles arabiensis	3	Opisthokonta
Echinococcus granulosus	3	Opisthokonta
Mucor circinelloides f. circinelloides 1006PhL	4	Fungi
Nematocida parisii ERTm1	4	Fungi
Encephalitozoon cuniculi GB−M1	4	Fungi
Smittium culicis	4	Fungi
Agaricus bisporus var. burnettii JB137−S8	5	Dikarya
Trametes cinnabarina	5	Dikarya
Cryptococcus gattii CA1280	5	Dikarya
Moesziomyces antarcticus	5	Dikarya
Microbotryum intermedium	5	Dikarya
Neolecta irregularis DAH−3	6	Ascomycota
Pneumocystis jirovecii RU7	6	Ascomycota
Protomyces lactucaedebilis	6	Ascomycota
Saitoella complicata NRRL Y−17804	6	Ascomycota
Schizosaccharomyces cryophilus OY26	6	Ascomycota
Bipolaris maydis ATCC 48331	7	Saccharomyceta
Zymoseptoria tritici ST99CH_1A5	7	Saccharomyceta
Acremonium chrysogenum ATCC 11550	7	Saccharomyceta
Neurospora tetrasperma FGSC 2508	7	Saccharomyceta
Blastomyces dermatitidis ATCC 18188	7	Saccharomyceta
Candida albicans SC5314	8	Saccharomycetales
Candida arabinofermentans NRRL YB−2248	8	Saccharomycetales
[Candida] auris	8	Saccharomycetales
Cyberlindnera fabianii	8	Saccharomycetales
Hanseniaspora guilliermondii	8	Saccharomycetales
Saccharomycetaceae sp. 'Ashbya aceri'	9	Saccharomycetaceae
Eremothecium gossypii ATCC 10895	9	Saccharomycetaceae
Kazachstania africana CBS 2517	9	Saccharomycetaceae
Kluyveromyces dobzhanskii CBS 2104	9	Saccharomycetaceae
Lachancea dasiensis CBS 10888	9	Saccharomycetaceae
Saccharomyces eubayanus	10	Saccharomyces
Saccharomyces uvarum	10	Saccharomyces
Saccharomyces arboricola	11	s5
Saccharomyces kudriavzevii	12	s4
Saccharomyces mikatae	13	s3
Saccharomyces paradoxus	14	s2
Saccharomyces cerevisiae	15	orphan
